# GIRK2 splice variants and neuronal G protein-gated K^+^ channels: implications for channel function and behavior

**DOI:** 10.1038/s41598-017-01820-2

**Published:** 2017-05-09

**Authors:** Ezequiel Marron Fernandez de Velasco, Lei Zhang, Baovi N. Vo, Megan Tipps, Shannon Farris, Zhilian Xia, Allison Anderson, Nicholas Carlblom, C. David Weaver, Serena M. Dudek, Kevin Wickman

**Affiliations:** 1 University of Minnesota, Department of Pharmacology, Minneapolis, MN 55455 USA; 2 National Institute of Environmental Health Sciences, Research Triangle Park, NC, 27709 USA; 3 Vanderbilt University, Department of Pharmacology, Nashville, TN 37235 USA

## Abstract

Many neurotransmitters directly inhibit neurons by activating G protein-gated inwardly rectifying K^+^ (GIRK) channels, thereby moderating the influence of excitatory input on neuronal excitability. While most neuronal GIRK channels are formed by GIRK1 and GIRK2 subunits, distinct GIRK2 isoforms generated by alternative splicing have been identified. Here, we compared the trafficking and function of two isoforms (GIRK2a and GIRK2c) expressed individually in hippocampal pyramidal neurons lacking GIRK2. GIRK2a and GIRK2c supported comparable somato-dendritic GIRK currents in *Girk2* ^*−/−*^ pyramidal neurons, although GIRK2c achieved a more uniform subcellular distribution in pyramidal neurons and supported inhibitory postsynaptic currents in distal dendrites better than GIRK2a. While over-expression of either isoform in dorsal CA1 pyramidal neurons restored contextual fear learning in a conditional *Girk2* ^*−/−*^ mouse line, GIRK2a also enhanced cue fear learning. Collectively, these data indicate that GIRK2 isoform balance within a neuron can impact the processing of afferent inhibitory input and associated behavior.

## Introduction

G protein-gated inwardly rectifying K^+^ (GIRK/Kir3) channels mediate the G protein-dependent, postsynaptic inhibitory effects of many neurotransmitters in the central nervous system, including GABA, dopamine, serotonin, acetylcholine, and glutamate^1^. GIRK channels are found almost exclusively in the somato-dendritic compartment and are enriched near excitatory synapses in spines^2^. Thus, these channels are well-positioned to temper the influence of excitatory input on neuronal activity^3^. Genetic and pharmacological investigations in mice have revealed important roles for GIRK channels in diverse settings, including seizures, learning/memory, nociception/analgesia, anxiety, and addiction^4–6^.

GIRK channels are homo- and heterotetrameric complexes formed by 4 subunits (GIRK1/Kir3.1, GIRK2/Kir3.2, GIRK3/Kir3.3, and GIRK4/Kir3.4)^2^. While GIRK1, GIRK2, and GIRK3 are widely expressed in the brain^7^, GIRK4 is found in only a few brain regions^8, 9^. Most, if not all, neuronal GIRK channels contain GIRK2^4^. Indeed, *Girk2* ^*−/−*^ mice exhibit small or absent synaptic and somato-dendritic GIRK currents in all neuron types evaluated to date^10–18^. GIRK1 cannot form functional homomeric channels, as it lacks an endoplasmic reticulum export signal^19–22^, and is thought to assemble with GIRK2 in most brain regions^23, 24^. Thus, the prototypical GIRK channel in the central nervous system is thought to contain both GIRK1 and GIRK2.

The diversity of neuronal GIRK channels is enhanced by alternative splicing which, in the case of GIRK2, yields at least 3 distinct proteins^22, 25–30^. Two GIRK2 variants (termed GIRK2a and GIRK2c) are particularly intriguing as they differ only by 11 amino acids in their intracellular C-terminal domains. GIRK2c is the longer of the 2 variants, and the 11 amino acid extension contains a conspicuous Class I PDZ interaction domain^22, 31^.

The impact of alternative splicing of the *Girk2* (*Kcnj6*) gene on GIRK channel trafficking and function is unclear. GIRK2c (but not GIRK2a) was reported to bind PSD-95 in one study^31^, but not in others^32, 33^. GIRK2c (but not GIRK2a) was also reported to bind to SAP-97, and co-expression of SAP-97 with GIRK2c conferred both basal activity and G protein-dependent gating to GIRK2c homomeric channels expressed in *Xenopus* oocytes^34^. To date, however, the attributes of GIRK2a and GIRK2c have not been systematically compared in mammalian cell systems. In this study, we expressed GIRK2a and GIRK2c independently in neurons lacking GIRK2, and compared their subcellular distributions and respective abilities to rescue GIRK channel function and GIRK-dependent behavior.

## Results

### Comparison of GIRK2a and GIRK2c-containing channels in transfected mammalian cells

**Figure 1:**
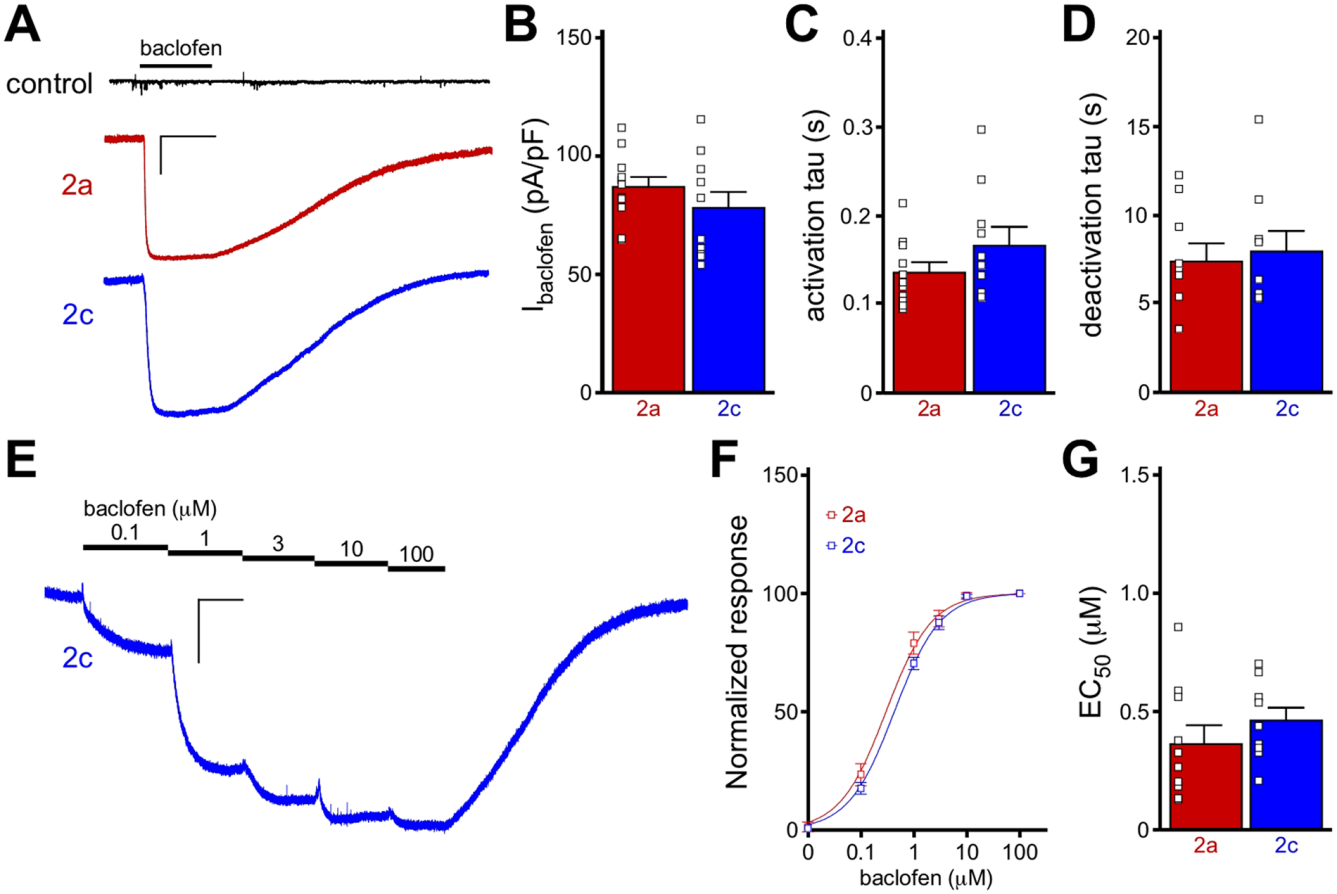
Functional comparison of GIRK2a and GIRK2c in HEK cells. (**A**) Whole-cell currents (V_hold_ = −70 mV) evoked by baclofen (100 μM) in HEK cells expressing GABA_B_R, GIRK1, and either GIRK2a (red) or GIRK2c (blue). No current was evoked by baclofen in cells expressing only GABA_B_R (control, black). Scale: 500 pA/10 s. (**B**) Summary of baclofen-induced, steady-state current densities (I_baclofen_, pA/pF) in HEK cells expressing GIRK1/GIRK2a or GIRK1/GIRK2c (*t*_19_ = 1.1, *P* = 0.29; n = 10–11/group). Individual data points are represented as small squares overlapping the relevant bar in the plot. (**C,D**) Activation (*t*_19_ = 1.5, *P* = 0.16) and deactivation (*t*_16_ = 0.4, *P* = 0.72) kinetics for baclofen-induced currents in HEK cells expressing GIRK1/GIRK2a or GIRK1/GIRK2c (n = 9–11/group). (**E**) Representative concentration-response experiment for a HEK cell expressing GABA_B_R and GIRK1/GIRK2c. Scale: 500 pA/10 s. (**F,G**) Summary of concentration-response experiments for baclofen-induced currents in HEK cells expressing GIRK1/GIRK2a or GIRK1/GIRK2c (*t*_17_ = 1.0, *P* = 0.32; n = 9–10/group). Currents were normalized to the response evoked by 100 μM baclofen in each experiment.

We began by comparing the functional properties of GIRK2a and GIRK2c expressed in mammalian (HEK) cells. As most neuronal GIRK channels are thought to be GIRK1/GIRK2 heteromers^23, 24^, HEK cells were transfected with GIRK1 and either GIRK2a or GIRK2c, along with the GABA_B_ receptor (GABA_B_R) subunits, GABA_B_R1 and GABA_B_R2. Whole-cell currents evoked by rapid bath application of the GABA_B_R agonist baclofen (100 μM) were measured (Fig. 1A). While no response to baclofen was seen with expression of GABA_B_R alone, cells expressing GABA_B_R and either GIRK1/GIRK2a or GIRK1/GIRK2c exhibited baclofen-induced currents of comparable size (Fig. 1A,B), with similar activation and deactivation kinetics (Fig. 1C,D). Furthermore, no difference in the EC_50_ for baclofen activation of GIRK1/GIRK2a or GIRK1/GIRK2c channels was observed (Fig. 1E–G), indicating that alternative splicing of GIRK2 does not impact the sensitivity of the prototypical neuronal GIRK channel to GABA_B_R activation in a typical mammalian expression system.

### GIRK2a and GIRK2c expression in the mouse hippocampus

**Figure 2:**
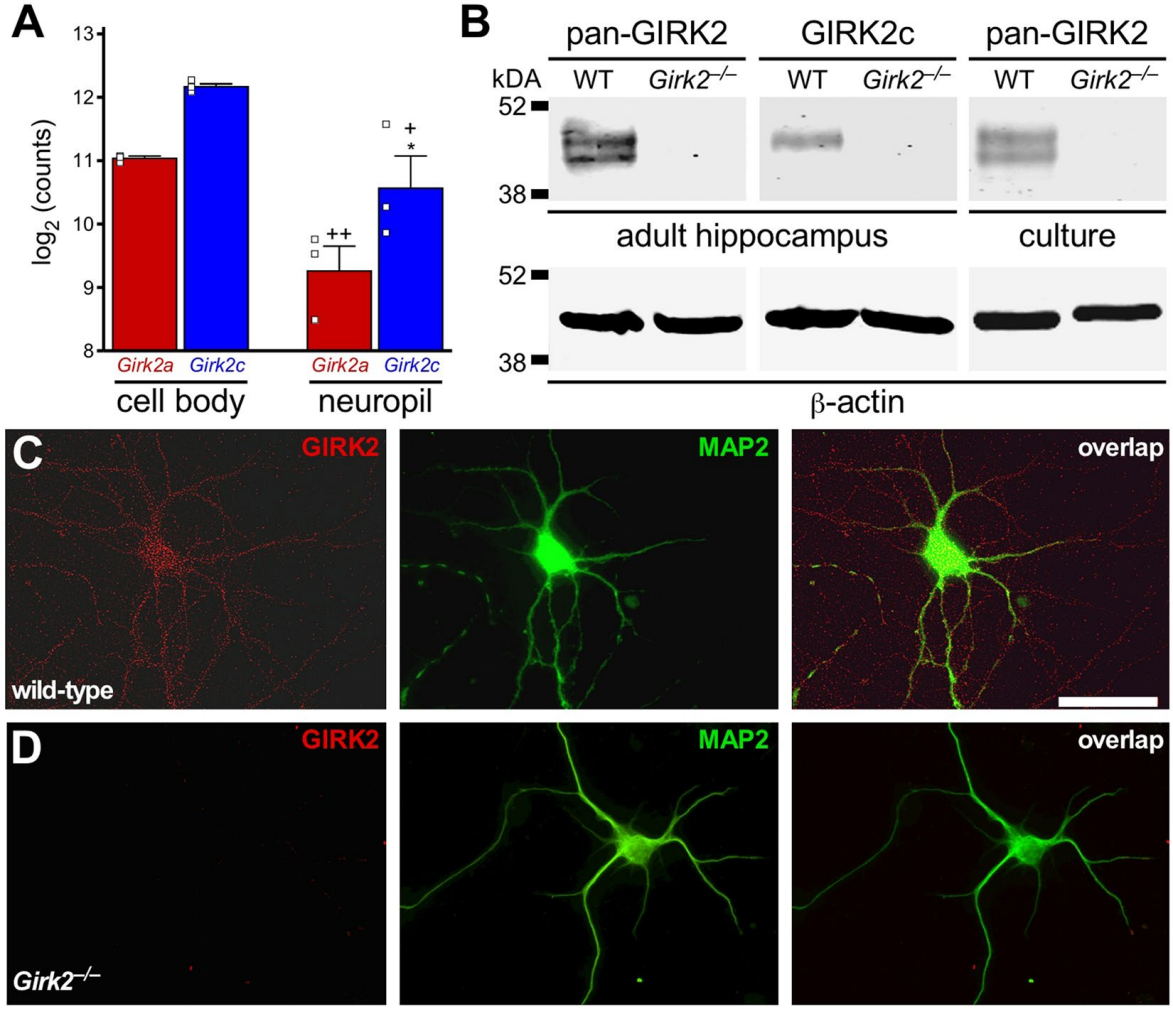
GIRK2 expression in the mouse hippocampus. (**A**) *Girk2a* and *Girk2c* mRNA levels as assessed by RNA-Seq in CA1 cell body and neuropil samples, taken from 3 adult mice. Main effects of isoform (F_1,8_ = 14.3, *P* < 0.01) and compartment (F_1,8_ = 27.1, *P* < 0.001) were observed, but there was no interaction between isoform and compartment (F_1,8_ = 0.07, *P* = 0.80). Symbols: *P < 0.05 vs. GIRK2a (within compartment); ^+,++^ *P* < 0.05 and 0.01, respectively, vs. cell body (within isoform). (**B**) Representative GIRK2 immunoblots (from 3 independent experiments) of adult hippocampus and neonatal hippocampal culture (10–12 DIV) from wild-type (WT) and *Girk2* ^*−/−*^ mice, probed with pan-GIRK2 or GIRK2c antibodies (upper blots), as well as a β-actin antibody (lower blots). (**C,D**) Representative images showing GIRK2 immunolabeling (as revealed with the pan-GIRK2 antibody) in cultured hippocampal neurons from wild-type (**C**) and *Girk2* ^*−/−*^ (**D**) mice. Neurons were co-stained with a MAP2 antibody to highlight neuronal morphology and dendritic processes. Scale bar: 50 microns.

HEK cells may lack factors that support GIRK2 isoform-specific contributions to channel trafficking and function, and thus, we next sought to compare the GIRK2 variants in a cell type in which they are normally expressed. Cell biological, biochemical, and electrophysiological studies involving wild-type and *Girk2* ^*−/−*^ mice have confirmed the presence of GIRK2 in mouse hippocampal neurons^10, 15^. Using RNA-Seq and laser-captured tissue samples from the adult mouse, we probed for *Girk2a* and *Girk2c* mRNAs in the CA1 region of the hippocampus. Because the 3′-UTR regions of *Girk2a* and *Girk2c* mRNAs are distinct and may influence their subcellular trafficking^29^, we compared transcript levels in cell body (*stratum pyramidale*) and neuropil (*stratum radiatum*) samples. Both transcripts were detected in neuropil, albeit at significantly lower levels than seen in CA1 cell body samples (q = 0.0012 for *Girk2a* and 0.0125 for *Girk2c*), suggesting that an active RNA trafficking mechanism exists for both isoforms (Fig. 2A). Higher levels of *Girk2c* mRNA relative to *Girk2a* mRNA were detected, with the difference reaching statistical significance in neuropil (*P* = 0.04) but not cell bodies (*P* = 0.07). Interestingly, we detected considerable read coverage past the annotated terminal exon for both transcripts, suggesting transcription occurs past the annotated 3′ ends in hippocampal pyramidal neurons.

We next assessed whether GIRK2a and GIRK2c proteins are present in mouse hippocampus and cultured hippocampal neurons. A commercially-available GIRK2 antibody (pan-GIRK2) targeting a domain in both isoforms revealed a doublet in intact hippocampus and cultured hippocampal neurons from wild-type but not *Girk2* ^*−/−*^ mice (Fig. 2B). We also used a custom GIRK2c-specific antibody, raised against the unique 11-amino acid domain of GIRK2c, to probe for the GIRK2c isoform in hippocampal samples. The GIRK2c antibody recognized GIRK2c, but not other GIRK subunits, when expressed in HEK cells (Supplementary Fig. S1). In hippocampal samples, this antibody revealed a single band that aligned with the upper band in the doublet seen with the pan-GIRK2 antibody (Fig. 2B). Thus, two GIRK2 splice isoform proteins are present in the mouse hippocampus, one definitively identified as GIRK2c and the other exhibiting an electrophoretic mobility consistent with that of GIRK2a.

### Subcellular distribution of GIRK2a and GIRK2c in *Girk2*^*−/−*^ pyramidal neurons

While GIRK2a and GIRK2c exhibited comparable labeling patterns when expressed in cultured rat hippocampal neurons^22^, endogenous GIRK2 might mask isoform-dependent differences in the trafficking and/or function of exogenously expressed subunits. Thus, we sought to examine the subcellular distribution of each isoform when expressed individually in cultured hippocampal neurons from *Girk2* ^*−/−*^ mice. AAV vectors harboring coding sequence for GIRK2a or GIRK2c, inserted downstream of the CaMKIIα promoter, were used to drive expression of untagged GIRK2a or GIRK2c (and EGFP, bi-cistronically) in *Girk2* ^*−/−*^ neurons. Strong overlap between recombinant GIRK2 and endogenous CaMKIIα immunolabeling was observed (Supplementary Fig. S2), confirming that the expression of GIRK2 was restricted primarily to pyramidal neurons.

**Figure 3:**
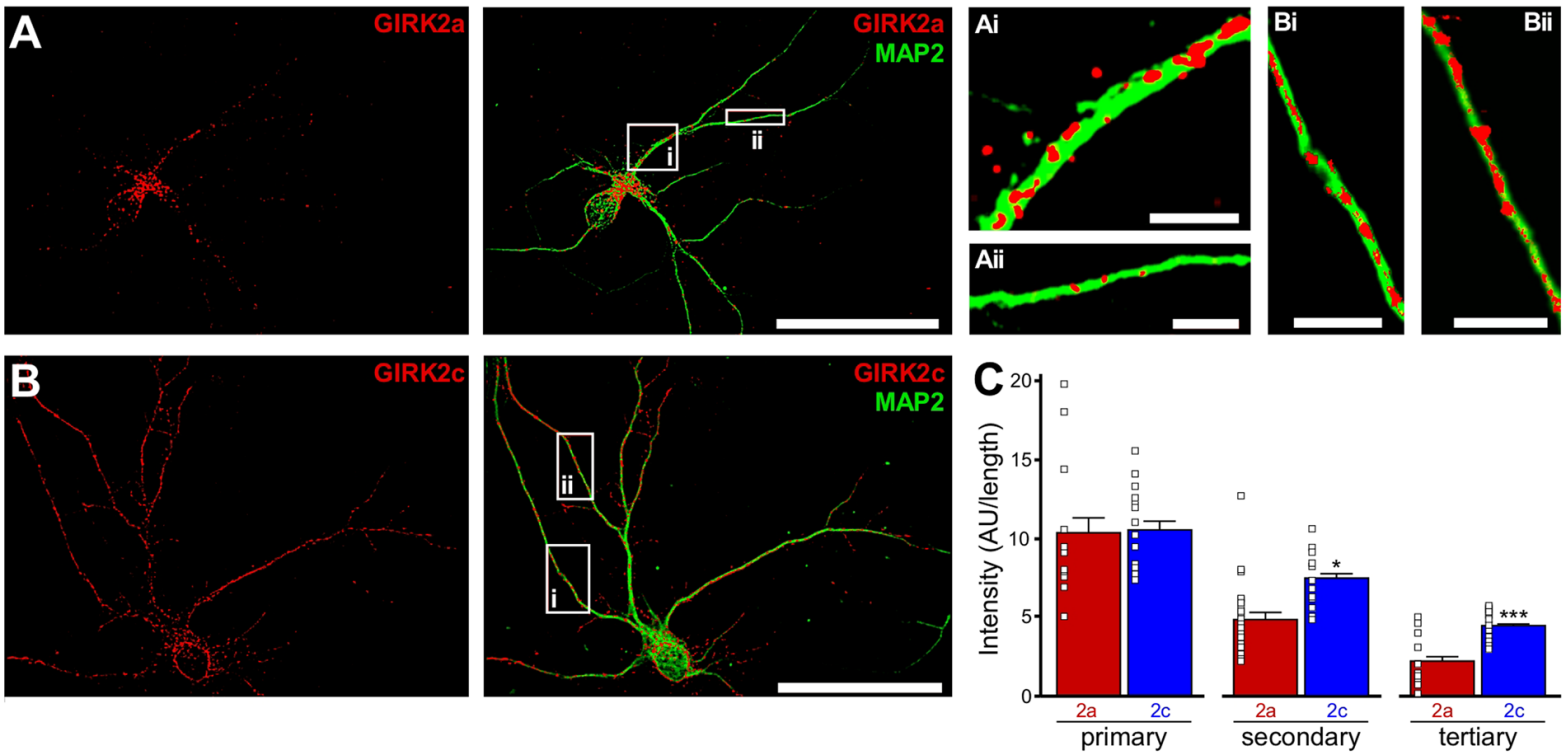
Subcellular distribution of GIRK2a and GIRK2c in *Girk2* ^*−/−*^ pyramidal neurons. (**A,B**) Representative images showing GIRK2 (red) and MAP2 (green) immunolabeling, and their overlay, in *Girk2* ^*−/−*^ hippocampal pyramidal neurons expressing either GIRK2a (**A**) or GIRK2c (**B**). Scale bars: 50 microns. The insets highlight different densities of GIRK2a and GIRK2c puncta along proximal/primary (Ai,Bi) and distal/secondary (Aii,Bii) dendritic segments. Scale bars: 5 microns. (**C**) Quantification of GIRK2a and GIRK2c labeling in dendrites from infected *Girk2* ^*−/−*^ pyramidal neurons. GIRK2 fluorescence intensity was measured in 2–3 primary (*t*_27_ = 1.4, *P* = 0.17), secondary (*t*_29_ = 2.1, *P < 0.05), and tertiary (*t*_27_ = 5.2, ***P < 0.001) dendritic segments from 5 different neurons expressing each subunit. Fluorescence intensity is expressed as arbitrary units (AU) normalized to segment length. Only GIRK2 labeling that overlapped directly with the dendritic segment (identified by MAP2 labeling) was quantified in this analysis.

Consistent with a previous report^35^, the pan-GIRK2 antibody yielded a punctate labeling pattern in wild-type cultures, with extensive labeling seen in dendritic processes (Fig. 2C). No such labeling was seen in hippocampal cultures from *Girk2* ^*−/−*^ mice (Fig. 2D), confirming antibody specificity. The labeling patterns for recombinant GIRK2a and GIRK2c expressed in *Girk2* ^*−/−*^ pyramidal neurons were overlapping but distinct (Fig. 3). While both subunits were detected in the soma and dendrites of infected neurons, GIRK2a labeling was more restricted to the cell body and proximal dendrites (Fig. 3A). In contrast, GIRK2c labeling was uniformly distributed throughout the neuron, including distal dendrites (Fig. 3B). Quantification of GIRK2 isoform labeling confirmed that GIRK2c exhibited a higher density of labeling than GIRK2a in secondary and tertiary dendrites of infected hippocampal pyramidal neurons (Fig. 3C).

GIRK3 has been shown to mediate interactions between GIRK channels and proteins that can regulate GIRK channel trafficking (sorting nexin 27, or SNX-27)^36^ and receptor coupling efficiency (regulator of G protein signaling 2, or RGS2)^37^. As GIRK3 is also expressed in hippocampal neurons^7, 15, 38^, we examined the subcellular distribution of GIRK2a and GIRK2c expressed in cultured hippocampal pyramidal neurons from mice lacking both GIRK2 and GIRK3 (*Girk2* ^*−/−*^ */Girk3* ^*−/−*^ mice). The labeling patterns for GIRK2a and GIRK2c in neurons from *Girk2* ^*−/−*^ */Girk3* ^*−/−*^ mice were comparable to those seen in *Girk2* ^*−/−*^ neurons (Supplementary Fig. S3), indicating that the GIRK2 isoform-dependent difference in subcellular distribution is not dependent on GIRK3.

### Overlap between PSD-95 and GIRK2 splice isoforms in *Girk2*^*−/−*^ pyramidal neurons

**Figure 4:**
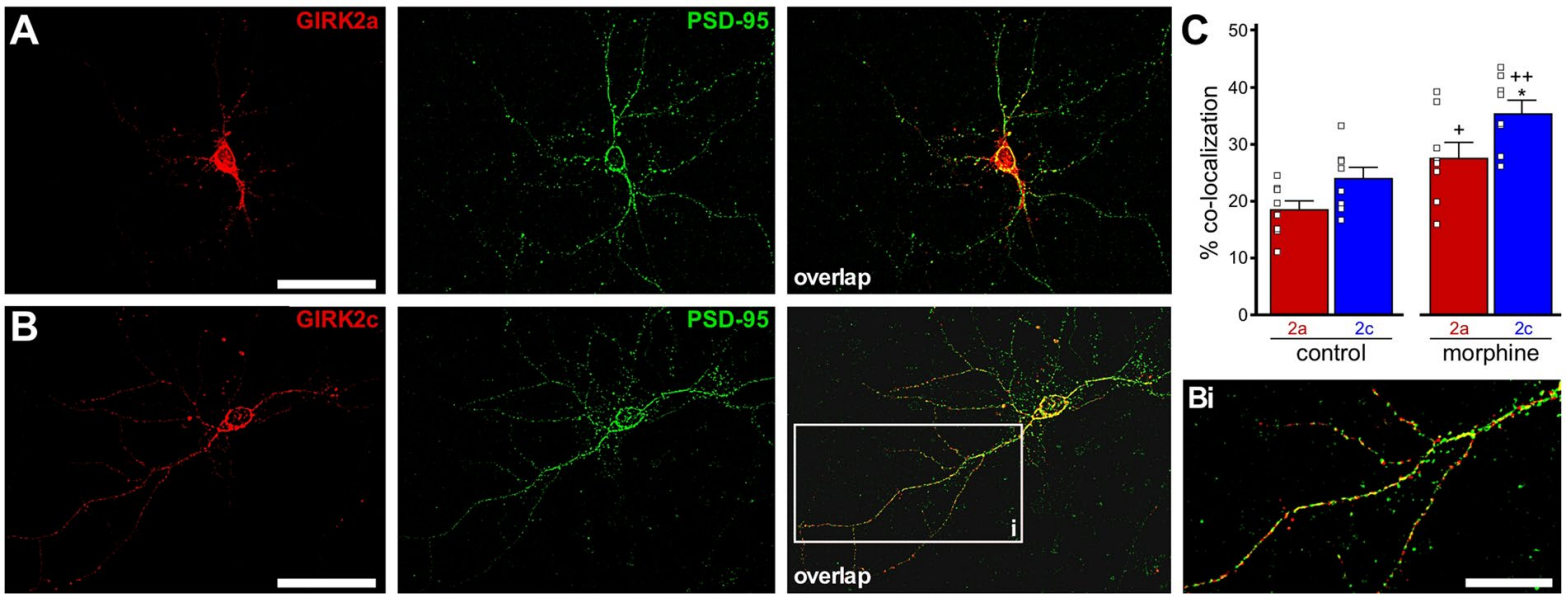
Overlap of GIRK2a and GIRK2c with PSD-95 in pyramidal neurons. (**A,B**) Representative images showing GIRK2 (red) and PSD-95 (green) immunolabeling, and their overlay, in a *Girk2* ^*−/−*^ hippocampal pyramidal neuron expressing GIRK2a (**A**) or GIRK2c (**B**). Scale bars: 50 microns. The inset (Bi) highlights the limited overlap between PSD-95 and GIRK2, as demonstrated with GIRK2c. Scale bars: 20 microns. (**C**) Quantification of overlap between PSD-95 and GIRK2a or GIRK2c, under control conditions and following morphine treatment (n = 8 per isoform and treatment condition). Main effects of isoform (F_1,28_ = 9.2, *P* < 0.01) and treatment (F_1,28_ = 22.1, *P* < 0.001) were observed, but there was no interaction between isoform and treatment (F_1,28_ = 0.3, *P* = 0.60). Symbols: *P < 0.05 vs. GIRK2a (within treatment); ^+,++^ *P* < 0.05 and 0.01, respectively, vs. control (within isoform).

GIRK2-containing channels are enriched near excitatory synapses^15, 39^, and some overlap between GIRK2 and PSD-95 was reported in cultured hippocampal neurons^35^. As the PDZ interaction motif found in GIRK2c may promote the association of GIRK channels with excitatory synapses, we next compared the overlap between endogenous PSD-95 and either GIRK2a or GIRK2c in *Girk2* ^*−/−*^ pyramidal neurons (Fig. 4A,B). No significant difference in the extent of overlap between PSD-95 and either GIRK2a or GIRK2c was detected at baseline (Fig. 4C, control). We also explored the possibility that incubation with morphine, a treatment previously shown to increase the overlap between GIRK2 and PSD-95 in cultured hippocampal neurons^35^, might reveal an isoform-dependent difference. Consistent with published observations, we found that morphine treatment increased total GIRK2 protein level by approximately 2-fold relative to vehicle-treated controls (Supplementary Fig. S4A,B), while having no apparent effect on PSD-95 labeling intensity (Supplementary Fig. S4C). Morphine treatment increased the overlap between PSD-95 and both GIRK2 isoforms relative to control conditions, with GIRK2c showing a small but significantly higher degree of overlap with PSD-95 than did GIRK2a following treatment (Fig. 4C, morphine; Supplementary Fig. S4D,E).

### GIRK currents measured in *Girk2*^*−/−*^ pyramidal neurons expressing GIRK2a or GIRK2c

**Figure 5:**
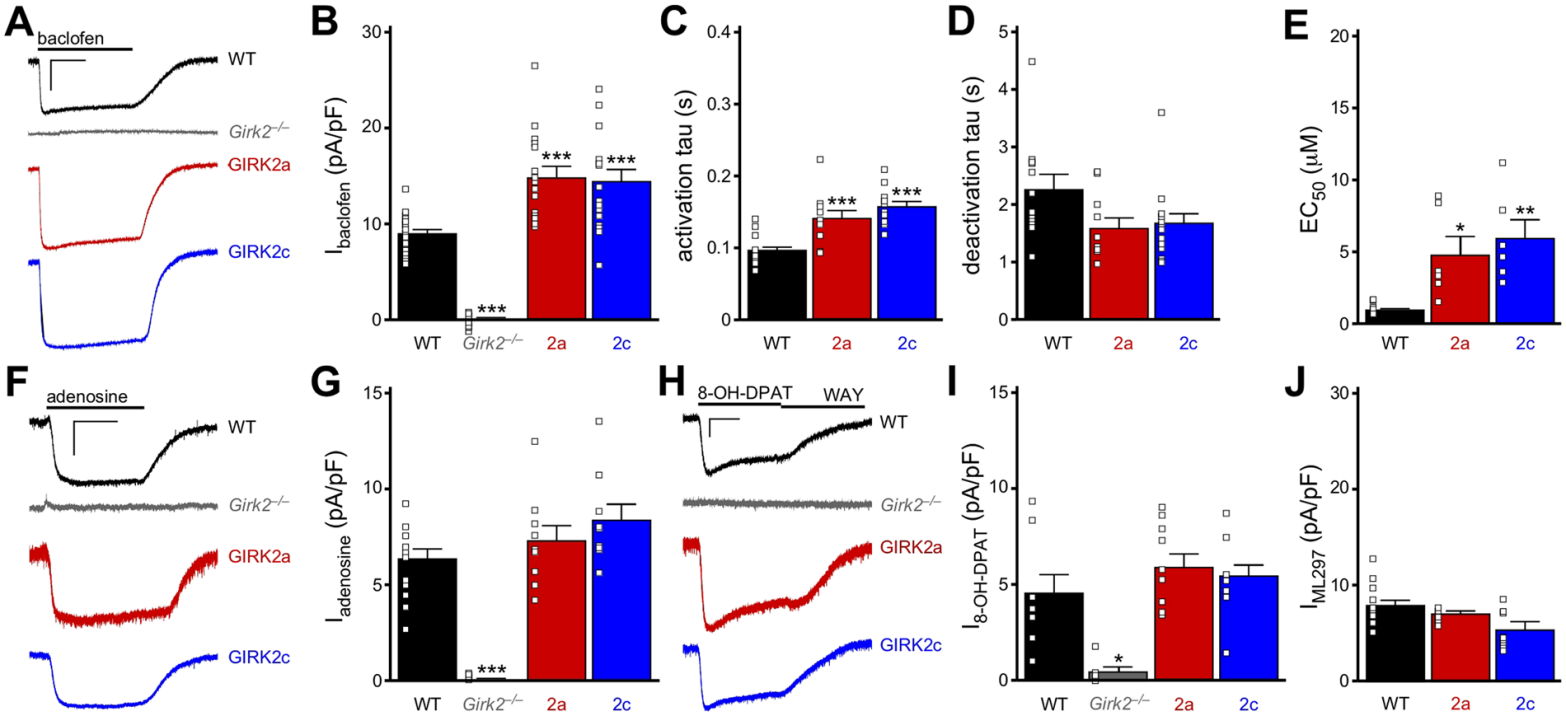
GPCR-GIRK currents in *Girk2* ^*−/−*^ pyramidal neurons expressing GIRK2a or GIRK2c. (**A**) Whole-cell currents (V_hold_ = −70 mV) evoked by baclofen (100 μM) in a wild-type pyramidal neuron expressing EGFP (WT, black), as well as *Girk2* ^*−/−*^ pyramidal neurons expressing EGFP (*Girk2* ^*−/−*^, gray), GIRK2a (red), or GIRK2c (blue). Scale: 1 nA/5 s. (**B**) Summary of baclofen-induced, steady-state current densities (pA/pF) in wild-type control and *Girk2* ^*−/−*^ pyramidal neurons expressing EGFP, GIRK2a, or GIRK2c (F_3,53_ = 44.8, *P* < 0.001; n = 11–16/group). Symbols: ***P < 0.001 vs. WT. (**C,D**) Summary of activation (C; F_2,35_ = 19.2, *P* < 0.001) and deactivation (**D**) F_2,33_ = 2.76, *P* = 0.08) kinetics for baclofen-induced currents in infected wild-type and *Girk2* ^*−/−*^ pyramidal neurons. Symbols: ***P < 0.001 vs. WT (n = 11–15 per group). (**E**) EC_50_ values for baclofen-induced currents in infected wild-type and *Girk2* ^*−/−*^ pyramidal neurons expressing GIRK2a or GIRK2c (F_2,17_ = 8.84, *P* < 0.01; n = 6–8/group). Symbols: *^,^**P < 0.05 and 0.01, respectively, vs. WT. (**F**) Whole-cell currents evoked by adenosine (10 μM) in a wild-type pyramidal neuron expressing EGFP, as well as *Girk2* ^*−/−*^ pyramidal neurons expressing EGFP, GIRK2a, or GIRK2c (V_hold_ = −70 mV). Scale: 500 pA/5 s. (**G**) Summary of adenosine-induced, steady-state current densities (I_adenosine_, pA/pF) in wild-type control and *Girk2* ^*−/−*^ pyramidal neurons expressing EGFP, GIRK2a, or GIRK2c (F_3,36_ = 32.1, *P* < 0.001; n = 9–13/group). Symbols: ***P < 0.001 vs. WT. (**H**) Whole-cell currents evoked by 8-OH-DPAT (10 μM) in a wild-type pyramidal neuron expressing EGFP, as well as *Girk2* ^*−/−*^ pyramidal neurons expressing EGFP, GIRK2a, or GIRK2c (V_hold_ = −70 mV). As currents evoked by 8-OH-DPAT persisted following bath washout, the 5HT_1A_R-selective antagonist WAY100635 (WAY, 2 μM) was used to demonstrate reversibility of the 8-OH-DAT response. Scale: 500 pA/5 s. (**I**) Summary of 8-OH-DPAT-induced, steady-state current densities (I_8-OH-DPAT_, pA/pF) in wild-type control and *Girk2* ^*−/−*^ pyramidal neurons expressing EGFP, GIRK2a, or GIRK2c (F_3,30_ = 7.92, *P* < 0.001; n = 5–11/group). Symbols: *P < 0.05 vs. WT. (**J**) Summary of current densities (pA/pF) evoked by 10 μM ML297 (I_ML297_) in wild-type control and *Girk2* ^*−/−*^ pyramidal neurons expressing EGFP, GIRK2a, or GIRK2c (F_2,12_ = 2.0, *P* = 0.16; n = 5–8/group).

We next asked whether GIRK2a and GIRK2c conferred unique functionality to GIRK channels in cultured hippocampal pyramidal neurons. Whole-cell currents evoked by GABA_B_R stimulation were measured in wild-type and *Girk2* ^*−/−*^ pyramidal neurons expressing EGFP, and in *Girk2* ^*−/−*^ pyramidal neurons expressing GIRK2a or GIRK2c. Baclofen evoked a prominent inward current in wild-type pyramidal neurons that was not seen in *Girk2* ^*−/−*^ control neurons expressing EGFP alone (Fig. 5A,B). Viral expression of either GIRK2 isoform in *Girk2* ^*−/−*^ pyramidal neurons yielded baclofen-induced currents that were larger than currents in wild-type controls. Activation (but not deactivation) kinetics of the baclofen-induced currents in *Girk2* ^*−/−*^ pyramidal neurons expressing GIRK2a or GIRK2c also differed from those in wild-type control neurons, though no isoform-dependent differences were found (Fig. 5C,D). Similarly, while there was no isoform-dependent difference in channel sensitivity (EC_50_) to GABA_B_R activation, GABA_B_R-GIRK coupling efficiency in both cases was diminished relative to wild-type controls (Fig. 5E).

GIRK channels in hippocampal pyramidal neurons are also activated by adenosine and serotonin^10, 40, 41^. To test whether GIRK2 isoforms promote preferential associations between GIRK channels and specific GPCRs, we measured GIRK currents evoked via these signaling pathways in *Girk2* ^*−/−*^ pyramidal neurons expressing GIRK2a or GIRK2c. Both adenosine (Fig. 5F) and the 5HT_1A_ receptor-selective agonist 8-OH-DPAT (Fig. 5H) evoked reliable currents in wild-type neurons, but not *Girk2* ^*−/−*^ pyramidal neurons expressing EGFP. *Girk2* ^*−/−*^ pyramidal neurons expressing either GIRK2a or GIRK2c exhibited adenosine- and 8-OH-DPAT-induced currents, and these currents were comparable in magnitude to those observed in wild-type control neurons (Fig. 5G,I). To gauge the influence of GIRK3 on the coupling between the GIRK2 isoforms and specific GPCRs, baclofen-, adenosine-, and 8-OH-DPAT-induced currents were also measured in infected *Girk2* ^*−/−*^ */Girk3* ^*−/−*^ pyramidal neurons. While no differences were found in *Girk2* ^*−/−*^ */Girk3* ^*−/−*^ pyramidal neurons expressing GIRK2a or GIRK2c, the currents measured in these neurons were larger than those recorded in *Girk2* ^*−/−*^ pyramidal neurons expressing these isoforms (Supplementary Fig. S5).

We also probed for differences in the interaction between the GIRK2 isoforms and endogenous GIRK1 in cultured *Girk2* ^*−/−*^ hippocampal neurons. Whole-cell currents were evoked by a selective activator of GIRK1-containing channels (ML297)^42, 43^. ML297-induced currents of comparable size were observed in *Girk2* ^*−/−*^ pyramidal neurons expressing GIRK2a or GIRK2c, and in wild-type control neurons (Fig. 5J), indicating that viral expression of either GIRK2 isoform restores a normal level of GIRK1-containing heteromeric channel activity.

### Synaptic GIRK currents in *Girk2*^*−/−*^ pyramidal neurons expressing GIRK2a or GIRK2c

**Figure 6:**
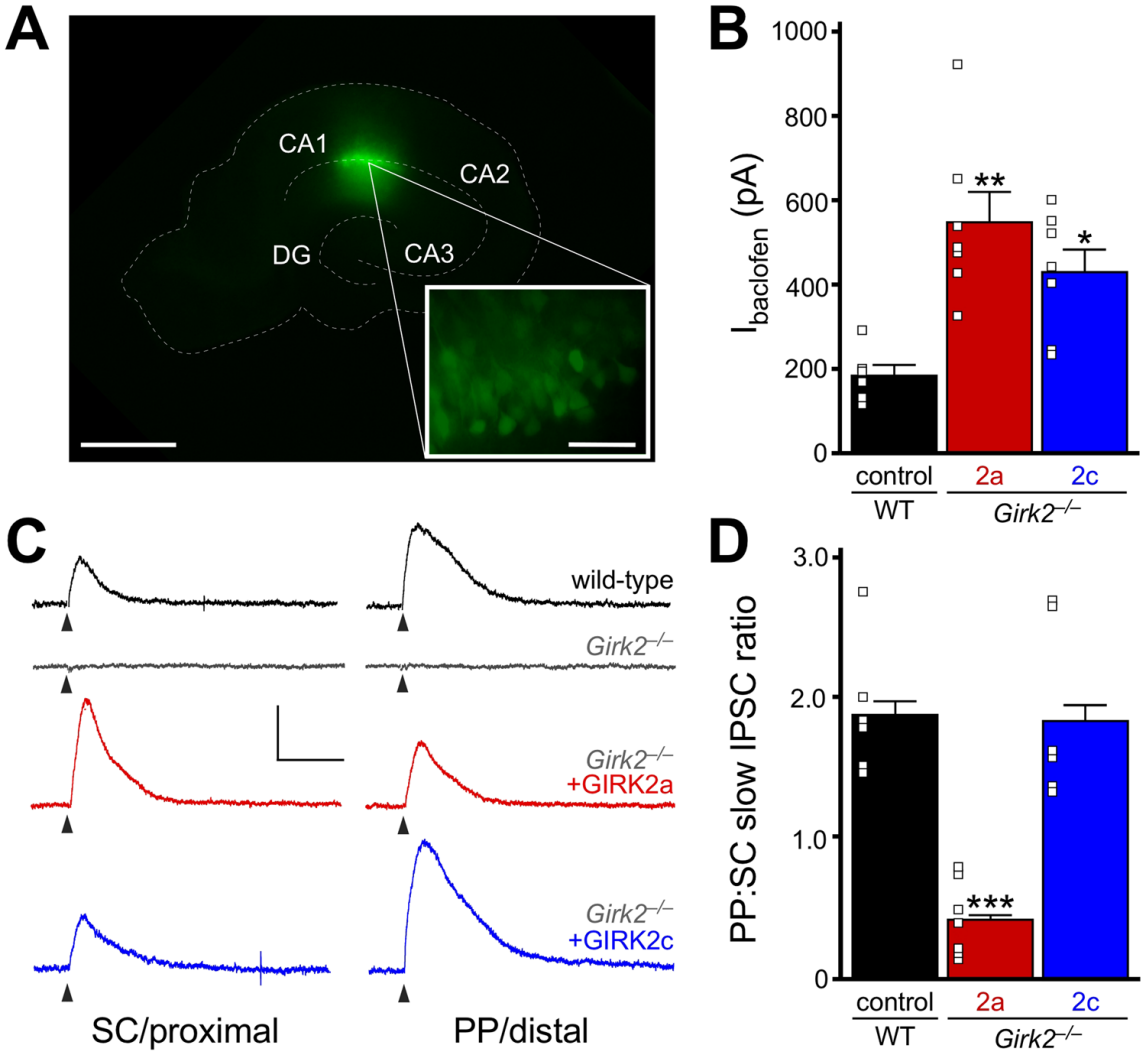
Synaptic GIRK currents in *Girk2* ^*−/−*^ neurons expressing GIRK2a and GIRK2c. (**A**) Image showing EGFP expression in the CA1 region of an organotypic hippocampal slice, taken 7 d after infection with AAV8-hSyn-GIRK2a-IRES-EGFP. The dotted white line highlights key features of slice morphology. Scale bar: 500 microns (inset: 50 microns). (**B**) Summary of peak baclofen-induced somatodendritic current amplitudes (I_baclofen_) in dorsal CA1 pyramidal neurons, measured 7 d after CA1 infusion of control virus (con) to wild-type (WT) organotypic slices, or GIRK2 expression viruses (2a, 2c) to *Girk2* ^*−/−*^ organotypic slices (F_2,17_ = 1.2, *P* < 0.01; n = 6–7/group). Symbols *^,^**P < 0.05 and 0.01, respectively, vs. WT. (**C**) Slow IPSCs evoked via stimulation of proximal (Schaffer collateral/SC) or distal (perforant path, PP) dendritic fields, in wild-type and *Girk2* ^*−/−*^ CA1 pyramidal neurons expressing EGFP, as well as *Girk2* ^*−/−*^ CA1 pyramidal neurons expressing GIRK2a or GIRK2c. The stimulus artifact and residual GABA_A_R-dependent responses were removed for clarity, and the triangles below the traces denote stimulation times. Scale: 100 pA/1 s. (**D**) Summary plot comparing the ratios of slow IPSC maximal amplitudes evoked by SC and PP stimulation in wild-type CA1 pyramidal neurons expressing EGFP, as well as *Girk2* ^*−/−*^ CA1 pyramidal neurons expressing GIRK2a or GIRK2c (F_2,37_ = 51.9; *P* < 0.001; n = 6–8/group). Maximal responses evoked by stimulation of SC and PP fields were measured in each neuron, in counterbalanced fashion. Symbols: ***P < 0.001 vs. WT (control) and *Girk2* ^*−/−*^ (GIRK2c).

The lack of natural connectivity and normal afferent inputs to cultured neurons complicates efforts to study the full range of physiological contributions made by GIRK channels to pyramidal neuron excitability. Thus, we next examined the functional properties of GIRK2a and GIRK2c in organotypic hippocampal slices. Organotypic hippocampal slices were cultured from young wild-type or *Girk2* ^*−/−*^ mice (P8–9), and the CA1 region was targeted with control or GIRK2 isoform-specific expression viruses (Fig. 6A). Following a 1-wk incubation period, we measured somatodendritic and synaptic GIRK-dependent responses in infected dorsal CA1 pyramidal neurons. Consistent with our observations in infected *Girk2* ^*−/−*^ pyramidal neurons in culture, we observed larger baclofen-induced somatodendritic currents in dorsal *Girk2* ^*−/−*^ CA1 pyramidal neurons expressing either GIRK2a or GIRK2c, as compared to wild-type controls (Fig. 6B **)**.

Slow inhibitory postsynaptic currents (slow IPSCs) in dorsal CA1 pyramidal neurons were evoked by electrical stimulation within proximal (Schaeffer collateral/SC) or distal (perforant path/PP) dendritic fields. SC and PP slow IPSCs were reliably observed in wild-type CA1 pyramidal neurons (Fig. 6C), and were blocked completely by the GABA_B_R receptor antagonist CGP54626 (2 μM, not shown). Slow IPSCs were not observed in *Girk2* ^*−/−*^ CA1 neurons expressing EGFP. While viral expression of either GIRK2a or GIRK2c in *Girk2* ^*−/−*^ slices restored both SC and PP slow IPSCs in CA1 pyramidal neurons, the relative amplitudes of slow IPSCs measured in SC and PP differed between the groups (Fig. 6C). Indeed, the ratio of slow IPSC amplitudes, measured in PP and SC for each neuron, was significantly smaller in *Girk2* ^*−/−*^ CA1 pyramidal neurons expressing GIRK2a, as compared to both wild-type control neurons, and *Girk2* ^*−/−*^ pyramidal neurons expressing GIRK2c (Fig. 6D).

### Fear conditioning in CaMKIICre(+):*Girk2*^*fl/fl*^ mice following GIRK2a or GIRK2c reconstitution

**Figure 7:**
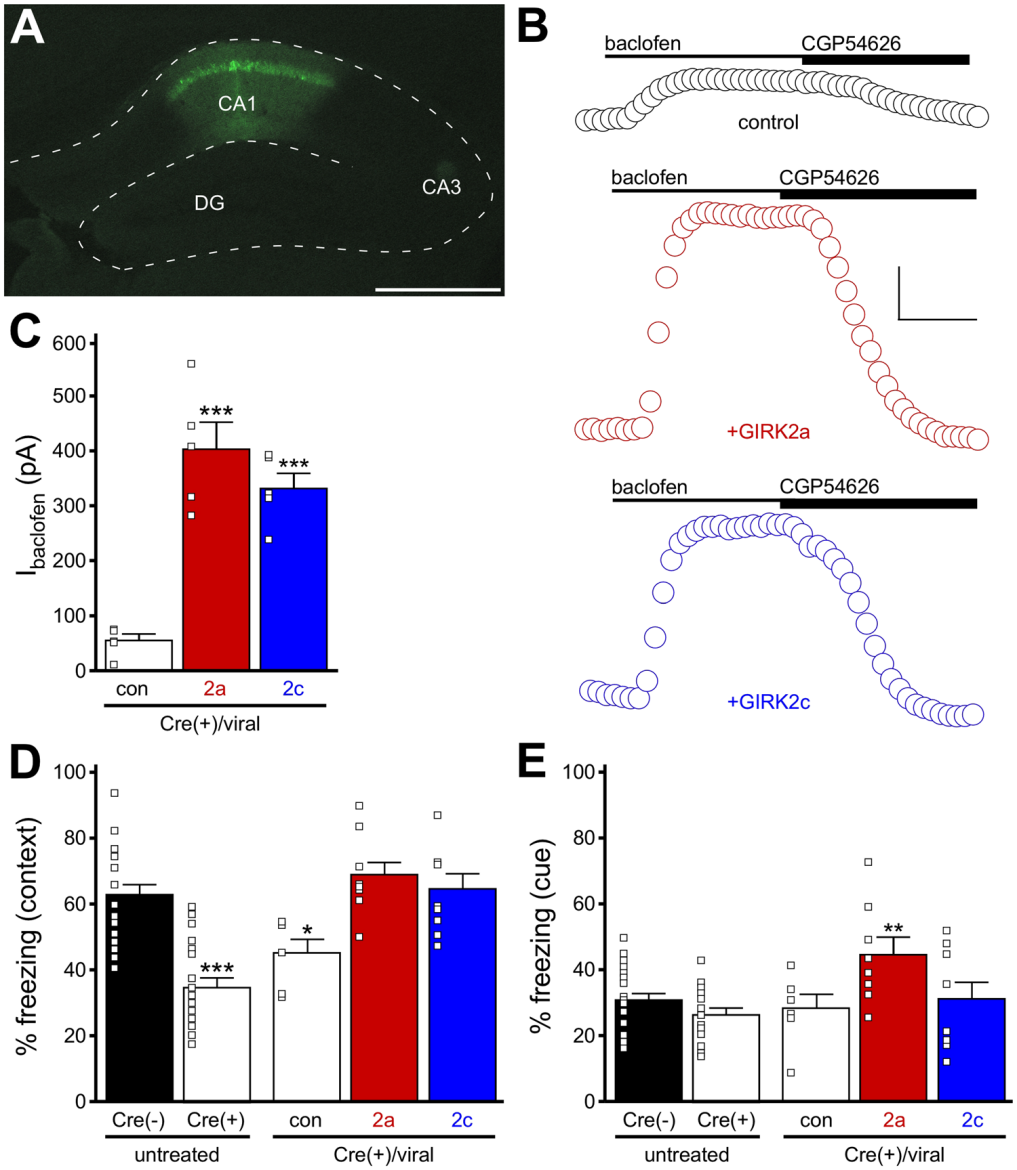
Fear learning in CaMKIICre(+):*Girk2* ^*fl/fl*^ mice expressing GIRK2a or GIRK2c. (**A**) EGFP expression in the dorsal CA1 of a CaMKIICre(+):*Girk2* ^*fl/fl*^ mouse, 2 wk after infusion of the AAV8-CaMKIIα-DIO-GIRK2a-IRES-EGFP virus. Scale bar: 500 microns. (**B**) Representative somato-dendritic currents (V_hold_ = −60 mV) evoked by baclofen (200 μM) and reversed by the GABA_B_R antagonist CGP54626 (2 μM) in dorsal CA1 pyramidal neurons from CaMKIICre(+):*Girk2* ^*fl/fl*^ mice, 2 wk after infusion of Cre-dependent control (con, mCherry), GIRK2a, or GIRK2c virus. Scale: 100 pA/100 s. (**C**) Summary of baclofen-induced currents in dorsal CA1 pyramidal neurons from CaMKIICre(+):*Girk2* ^*fl/fl*^ mice, 2 wk after infusion of Cre-dependent control (con, mCherry), GIRK2a, or GIRK2c virus (F_2,11_ = 24.3, *P* < 0.001; n = 4–5/group). Symbols: ***P < 0.001 vs. control. (**D**) Contextual fear learning in untreated CaMKIICre(−):*Girk2* ^*fl/fl*^ mice and CaMKIICre(+):*Girk2* ^*fl/fl*^ mice, and in CaMKIICre(+):*Girk2* ^*fl/fl*^ mice following infusion of Cre-dependent control (con, mCherry), GIRK2a, or GIRK2c virus in the dorsal CA1 (F_4,55_ = 14.7, *P* < 0.001; n = 6–23/group). Symbols: *^,^***P < 0.05 and 0.001, respectively, vs. Cre(−). (**E**) Cue fear learning in untreated CaMKIICre(−):*Girk2* ^*fl/fl*^ mice and CaMKIICre(+):*Girk2* ^*fl/fl*^ mice, and in CaMKIICre(+):*Girk2* ^*fl/fl*^ mice following infusion of Cre-dependent control (con, mCherry), GIRK2a, or GIRK2c virus in the dorsal CA1 (F_4,55_ = 4.0, *P* < 0.01; n = 6–23/group). Symbols: **P < 0.01 vs. Cre(−).

Recently, we reported that mice lacking GIRK2 in forebrain pyramidal neurons (CaMKIICre(+):*Girk2* ^*fl/fl*^ mice) were deficient in contextual fear learning, a hippocampal-dependent behavior^6^. As CaMKIICre(+):*Girk2* ^*fl/fl*^ mice exhibited a loss of GIRK-dependent signaling in dorsal but not ventral CA1 pyramidal neurons^6^, we asked whether viral restoration of GIRK2a or GIRK2c in dorsal CA1 pyramidal neurons of CaMKIICre(+):*Girk2* ^*fl/fl*^ mice could rescue normal fear learning. Adult CaMKIICre(+):*Girk2* ^*fl/fl*^ mice received bilateral infusions of Cre-dependent variants of the GIRK2a and GIRK2c expression viruses into the dorsal CA1 (Fig. 7A). After a suitable post-surgical recovery period (2 wk), we tested the efficacy of our viral treatment by measuring baclofen-induced somato-dendritic currents in CA1 pyramidal neurons in acutely isolated slices. Currents in dorsal CA1 pyramidal neurons from CaMKIICre(+):*Girk2* ^*fl/fl*^ mice treated with control virus were small (Fig. 7B,C), and comparable in size to those reported previously for untreated CaMKIICre(+):*Girk2* ^*fl/fl*^ mice^6^. Dorsal CA1 pyramidal neurons from CaMKIICre(+):*Girk2* ^*fl/fl*^ mice expressing GIRK2a or GIRK2c exhibited significantly larger baclofen-induced currents (Fig. 7B,C). Indeed, currents measured in dorsal CA1 pyramidal neurons expressing GIRK2a or GIRK2c were notably larger than those reported previously in wild-type or CaMKIICre(−):*Girk2* ^*fl/fl*^ controls^6, 15^, suggesting that the GIRK2 isoforms were over-expressed in these neurons.

We utilized a trace fear conditioning protocol to assess the impact of viral treatment on fear learning behavior, as both contextual and cue fear learning in this model are supported by the dorsal hippocampus^44, 45^. Untreated CaMKIICre(+):*Girk2* ^*fl/fl*^ mice, as well as CaMKIICre(+):*Girk2* ^*fl/fl*^ mice treated with control virus, exhibited a deficit in contextual fear learning in this model (Fig. 7D), consistent with the contribution of GIRK-dependent signaling in dorsal CA1 to this behavior. Over-expression of either GIRK2a or GIRK2c in dorsal CA1 pyramidal neurons restored contextual fear learning in CaMKIICre(+):*Girk2* ^*fl/fl*^ mice (Fig. 7D). Interestingly, while untreated CaMKIICre(+):*Girk2* ^*fl/fl*^ mice, and CaMKIICre(+):*Girk2* ^*fl/fl*^ mice treated with control virus, exhibited normal cue fear learning, over-expression of GIRK2a but not GIRK2c in dorsal CA1 pyramidal neurons from CaMKIICre(+):*Girk2* ^*fl/fl*^ mice significantly enhanced cue fear learning (Fig. 7E). Thus, GIRK2a and GIRK2c exert overlapping but distinct influences on dissociable forms of hippocampal-dependent fear learning.

## Discussion

In this study, we probed for differences between GIRK2a and GIRK2c in an array of expression systems. Our findings suggest that these two isoforms differ primarily in terms of subcellular trafficking, with GIRK2c achieving a more uniform distribution throughout neurons, including distal dendrites. This difference in subcellular trafficking likely underlies the differing abilities of GIRK2a and GIRK2c to support slow IPSCs evoked via the electrical stimulation of proximal and distal dendritic fields. Indeed, our findings suggest that GIRK2c is critical for ensuring robust synaptic inhibitory GIRK-dependent responses in the distal dendritic fields of CA1 pyramidal neurons.

We observed some interesting differences between GIRK-dependent signaling in transfected HEK cells and infected *Girk2* ^*−/−*^ pyramidal neurons. Notably, GIRK1/GIRK2 channels expressed in HEK cells were more sensitive to GABA_B_R activation (*i.e*., they exhibited a lower EC_50_) than the GIRK channels found in wild-type pyramidal neurons or *Girk2* ^*−/−*^ neurons expressing GIRK2a or GIRK2c. This difference could be explained by a higher density of receptor and channel expressed in HEK cells, and/or by different receptor-channel ratios achieved in the different cell systems. The difference in coupling efficiency could also reflect the presence of negative regulatory elements in neurons that are not found in HEK cells. Indeed, we reported previously that a complex consisting of RGS7 and Gβ5 decreases the sensitivity of GIRK channels to GABA_B_R activation in hippocampal neurons^46–48^, while also prominently accelerating GABA_B_R-GIRK current deactivation kinetics. In support of this contention, the deactivation kinetics of GABA_B_R-GIRK currents were significantly faster in neurons than in HEK cells.

Another difference between HEK cell and neuron models is the presence of GIRK3, which has been reported to decrease the activity and surface trafficking of GIRK1/GIRK2 heteromers and GIRK2 homomers in expression systems^22, 49^. We show here that while the relative dendritic labeling patterns for GIRK2a and GIRK2c were comparable in *Girk2* ^*−/−*^ and *Girk2* ^*−/−*^ */Girk3* ^*−/−*^ neurons, GPCR-GIRK currents measured in *Girk2* ^*−/−*^ */Girk3* ^*−/−*^ neurons expressing GIRK2a or GIRK2c were larger than those measured in *Girk2* ^*−/−*^ neurons. These results are consistent with the possible role of GIRK3 as a negative regulator of GIRK channel surface trafficking. In midbrain DA neurons, GIRK3 mediates an interaction between the GIRK channel, a GIRK2c/GIRK3 heteromer^13^, and SNX-27^36, 50, 51^, a protein implicated in the surface trafficking and internalization of several channels and receptors^52–56^. The interaction between SNX-27 and GIRK3 is conferred by a Class I PDZ interaction domain at the GIRK3 C-terminus, which is identical to that found in GIRK2c^33^. At present, the relevance of SNX-27 and GIRK3 to GIRK channel trafficking in hippocampal neurons is unclear.

Previous immunoelectron microscopy studies have shown that GIRK1 and GIRK2 labeling is concentrated at the peri- and extra-synaptic positions relative to the postsynaptic density (PSD) in hippocampal CA1. In addition, GIRK2 (but not GIRK1) labeling was detected within the PSD of CA1 pyramidal neurons^15^. The presence of a PDZ domain on the C-terminus of GIRK2c suggested that this isoform might promote the distribution of GIRK channels within or around the PSD, by virtue of its interaction with a scaffolding protein such as PSD-95. As noted above, GIRK2c was reported to bind PSD-95^31^, as well as SAP-97^34^. Moreover, SAP97 conferred both basal activity and G protein-dependent gating to GIRK2c-containing channels expressed in *Xenopus* oocytes^34^. Our data here, however, show that GIRK2a and GIRK2c assume comparable subcellular distributions relative to PSD-95 in mouse hippocampal pyramidal neurons. Thus, alternative splicing of the coding sequence of the *Girk2* gene does not seem to influence the position of GIRK channels relative to the PSD. We did, however, detect significantly more *Girk2c* than *Girk2a* mRNA in the neuropil of CA1 *in vivo*, suggesting that RNA localization may play a role in setting the amount of GIRK2 isoforms in CA1 dendrites. Moreover, our RNA-Seq data suggest that the 3′ terminal exons of both isoforms may be extended *in vivo*, which could further regulate the targeting, translation, and turnover of these transcripts. Follow up studies looking at the significance of this extended transcription are needed.

The level of GIRK1 protein is significantly reduced in the hippocampus of constitutive *Girk2* ^*−/−*^ mice^15, 24^. Our experiments with the GIRK1-selective agonist ML297, however, suggest that viral expression of GIRK2 isoforms in *Girk2* ^*−/−*^ pyramidal neurons restored a normal level of functional GIRK1/GIRK2 heteromeric channels. Baclofen-induced currents in *Girk2* ^*−/−*^ pyramidal neurons expressing either GIRK2a or GIRK2c were significantly larger than those measured in wild-type controls, however, indicating that some of the GABA_B_R-GIRK current was carried by GIRK2 homomeric or GIRK2/GIRK3 heteromeric channels. Interestingly, expression of GIRK1 in VTA dopamine neurons, which normally express a GIRK2c/GIRK3 heteromeric channel, increased GIRK channel sensitivity to GABA_B_R activation^37^. Moreover, GIRK2/GIRK3 heteromers are less sensitive to G protein activation than GIRK2 homomers^37^. Thus, an altered proportion of GIRK1-containing and GIRK1-lacking channels in *Girk2* ^*−/−*^ pyramidal neurons expressing GIRK2a or GIRK2c could explain why some features of the baclofen-induced current, including the EC_50_, differed from those in wild-type neurons. Notably, adenosine and 5HT_1A_R-dependent currents in *Girk2* ^*−/−*^ neurons expressing GIRK2a or GIRK2c did not differ in terms of amplitude from wild-type controls, suggesting that adenosine and 5HT_1A_ receptors may couple preferentially to GIRK1-containining channels, while GABA_B_R may be able to couple effectively to GIRK channels irrespective of channel subtype.

Multiple forms of plasticity involving GIRK-dependent signaling in hippocampal neurons have been reported. For example, Jan and colleagues showed that stimuli capable of inducing long-term potentiation (LTP) also enhanced GABA_B_R-GIRK signaling in CA1 pyramidal neurons^57^. Subsequently, this group showed that NMDA receptor activation triggered a rapid increase in surface trafficking of GIRK channels in hippocampal cultures^58, 59^. This adaptation involved the dephosphorylation of an N-terminal GIRK2 residue (Ser-9) found in both GIRK2a and GIRK2c, and yielded enhanced basal GIRK channel activity and GIRK channel responses to adenosine receptor activation, but had no effect on GABA_B_R-GIRK signaling. Slesinger and colleagues showed that prolonged morphine treatment in hippocampal cultures shifted the subcellular distribution of GIRK2 from dendritic shafts to spines, leading to enhanced basal and 5HT-induced GIRK currents, but no change in GABA_B_R-GIRK signaling^35^. Our data suggest that this morphine-induced adaptation does not require elements within the *Girk2* promoter or untranslated domains in the *Girk2* mRNAs, as only coding sequence was included in the viral reconstitution vectors. The unique GIRK2c C-terminus is also not required, as both GIRK2a and GIRK2c were similarly impacted by morphine treatment. Interestingly, the strengthening of GIRK-dependent signaling in VTA dopamine neurons evoked by burst firing was blocked by inclusion of a peptide mimic of the GIRK2c (and GIRK3) C-terminal domain^60^. These data indicate that the GIRK2c C-terminal domain may be a critical determinant of plasticity involving specific GIRK channel subtypes, including the GIRK1-lacking channels expressed in midbrain dopamine neurons^13, 31^.

Constitutive *Girk2* ^*−/−*^ mice exhibit an array of neurological phenotypes, including impaired fear learning^6, 61^. The global nature of the genetic manipulation, however, complicates attempts to link the phenotype to a defined neuron population. Accordingly, we developed conditional *Girk2* ^*−/−*^ (*Girk2* ^*fl/fl*^) mice and have crossed them with transgenic Cre driver lines to permit GIRK channel ablation in a neuron-specific manner^6, 61^. In CaMKIICre(+):*Girk2* ^*fl/fl*^ mice, *Girk2* ablation is driven by Cre recombinase, expressed under the control of the CaMKIIα promoter. While the CaMKIICre driver line is generally considered to promote gene ablation in postnatal forebrain pyramidal neurons, the scope of recombination achieved with this driver line depends on the “floxed” gene in question, with some studies showing highly restricted target loss in dorsal CA1 pyramidal neurons and others showing recombination in multiple forebrain pyramidal neuron populations^62–67^. We found that GIRK2 expression was preserved in most forebrain structures in CaMKIICre(+):*Girk2* ^*fl/fl*^ mice, but that GIRK-dependent signaling was blunted in dorsal (but not ventral) CA1 pyramidal neurons^6^.

To further probe the contribution of GIRK-dependent signaling in the hippocampus to fear learning, we assessed the performance of CaMKIICre(+):*Girk2* ^*fl/fl*^ mice in trace fear conditioning test. In this paradigm, the trace interval inserted between the presentation of the conditioned (cue/tone) and unconditioned (footshock) stimulus activates temporal memory mechanisms within the hippocampus, thus rendering both contextual and cue fear learning dependent on the hippocampus^44, 68^. Viral-mediated over-expression of either GIRK2a or GIRK2c in dorsal CA1 pyramidal neurons rescued contextual fear learning in CaMKIICre(+):*Girk2* ^*fl/fl*^ mice, suggesting that the fear learning deficit in CaMKIICre(+):*Girk2* ^*fl/fl*^ mice is linked to GIRK2 loss in these neurons. Although we did not see a difference in cue fear learning between untreated CaMKIICre(−):*Girk2* ^*fl/fl*^ and CaMKIICre(+):*Girk2* ^*fl/fl*^ mice, over-expression of GIRK2a, but not GIRK2c, in dorsal CA1 pyramidal neurons significantly enhanced cue learning. The enhanced cue learning seen in CaMKIICre(+):*Girk2* ^*fl/fl*^ mice over-expressing GIRK2a in dorsal CA1 pyramidal neurons, together with our evidence that the GIRK2 isoforms contribute in distinct fashion to the processing of inhibitory input to proximal and distal dendritic fields, suggests that trace cue learning may be differentially modulated by inputs from the PP and SC.

In summary, we demonstrate in this study that alternative splicing of the mouse *Girk2* gene generates at least two variants, and that these splice variants can give rise to channel subtypes that differ in terms of their subcellular trafficking and the neurophysiological processes in which they participate. As GIRK-dependent signaling plays an important role in moderating glutamatergic input to neurons, our findings raise the intriguing possibility that altering the balance of GIRK2 splice variants could represent a means by which neurons fine-tune the processing of excitatory synaptic input, in a subcellular compartment-dependent manner.

## Methods

### Animals

All animal studies were approved by the Institutional Animal Care and Use Committee at the University of Minnesota or NIEHS, and were conducted in accordance with National Institutes of Health guidelines for the care and use of animals in research. The generation of *Girk2* ^*−/−*^, *Girk2* ^*−/−*^ */Girk3* ^*−/−*^, and CaMKIICre(+):*Girk2* ^*fl/fl*^ mice was described previously^6, 12, 24^. The Amigo2-EGFP line was acquired from GENSAT (founder line LW244), and bred for at least 10 generations onto C57BL6/n background. Mice were maintained on a 12 h light/dark cycle (lights on at 0700), with food and water available *ad libitum*.

### Chemicals

Baclofen (*R*-(+)-β-(aminomethyl)-4-chlorobenzenepropanoic acid hydrochloride), adenosine, CGP54626, kynurenic acid, and picrotoxin were purchased from Sigma-Aldrich (St. Louis, MO). (R)-(+)-8-OH-DPAT hydrobromide and WAY100635 maleate were purchased from Tocris (Bristol, UK). ML297 was synthesized in the Vanderbilt Center for Neuroscience Drug Discovery.

### DNA constructs and virus production

pcDNA3-based expression constructs containing coding sequence for rat GIRK1, mouse GIRK2a (NM_001025584.2, initially referred to as Girk2-1: NP_001020755, AAC34284), and mouse GIRK2c (NM_010606.2, initially referred to as Girk2-A or Girk2A-1: NP_034736, AAC34285, P48542), as well as mouse GABA_B_R1 and GABA_B_R2 (provided by Dr. Paul Slesinger), were used for HEK293 cell transfection and electrophysiology studies. AAV vectors used for expression of GIRK2a or GIRK2c were generated by sub-cloning GIRK2a/c-IRES-EGFP cassettes into the backbones of pAAV-hSyn-TRβ-IRES-EGFP (provided by Dr. Bernd Gloss), pAAV-CaMKIIα-hChR2(C128S/D156A)-mCherry (provided by Dr. Karl Deisseroth; Addgene plasmid #35502)^69^, and pAAV-hSyn-DIO-hM4D(Gi)-mCherry (provided by Dr. Bryan Roth; Addgene plasmid #44362)^70^ plasmids. The AAV Helper-Free System was used for viral packaging (Agilent Technologies; Santa Clara, CA). AAV particles were obtained by co-transfecting pHelper (provided by Dr. David Armstrong), pAAV-RC2/8 (UPenn Viral Vector Core), and pShuttle plasmids into AAV293 cells using the polyethylenimine (PEI) method (Polysciences, Inc.; Warrington, PA). For each 15-cm plate, a total of 100 μg of DNA (33.3 μg of each plasmid) was added to a 150 mM NaCl solution, in a final volume of 2 mL; 12.5 μL of PEI (16 mg/ml) was then added while mixing constantly to favor the formation of DNA/PEI complexes. After 10 min of incubation, the complexes were added drop wise to AAV293 cells maintained in DMEM supplemented with 10% FBS, 4 mM L-glutamine, 1 mM sodium pyruvate at 37 °C and 5% CO_2_. After 72 h, cells were collected for viral particle extraction and purification. Viral titering was performed by qPCR with specific primers for the WPRE region of the AAV vectors: 5′-CCGTTGTCAGGCAACGTG-3′ (forward) and 5′-AGCTGACAGGTGGTGGCAAT-3′ (reverse). Control viruses (AAV8-CaMKII-EGFP, AAV8-hSyn-EGFP, and AAV8-hSyn-DIO-mCherry) were obtained from the UNC Viral Vector Core (Chapel Hill, NC).

### RNA-Seq and analysis

Total RNA was extracted from laser-captured tissue microdissected from hippocampal CA1 cell body and neuropil layers from 3 adult (6–8 wk) male Amigo2-EGFP transgenic mice using the micro RNeasy kit (QIAGEN; Hilden, Germany), including on-column DNase-treatment. Total RNA samples were analysed for RNA integrity (RIN > 8.5) and concentration using the 2100 Bioanalyzer instrument (Agilent; Santa Clara, CA) and RNA 6000 Pico assay (Agilent). Stranded RNA-Seq libraries were prepared using 1–5 ng of total RNA per sample and region (1 ng for neuropil samples and 5 ng for cell body samples) with the Ovation RNA-Seq Systems 1–16 for mouse (NuGEN; San Carlos, CA), according to manufacturer instructions. Libraries were analyzed for size and concentration using the 2100 Agilent Bioanalyzer and the High Sensitivity DNA assay. Libraries were multiplexed and run on a NextSeq500 instrument (Illumina; San Diego, CA), acquiring an average of 66 ± 17 million 100 bp reads per sample. Reads were trimmed using Sickle software (https://github.com/najoshi/sickle) and only paired reads with a quality score >20 and a minimum length of 20 bp were pseudo-aligned and quantified to merged GENCODE (M9) and RefSeq gene models using Kallisto^71^. Samples were normalized and pairwise comparisons (cell body to neuropil) were made using Sleuth software (https://github.com/pachterlab/sleuth)^72^, with a false discovery rate (FDR, q value) of 0.05 (reported in *Results*). Scatter plots for *Kncj6* transcripts corresponding to accession numbers NM_001025584.2 (*Girk2a*) and NM_010606.2 (*Girk2c*) were made using log_2_ normalized counts.

### Cell culture and transfection/infection

HEK293 cells (ATCC; Manassas, VA) were cultured at 37 °C/5%CO_2_ according to supplier recommendations. For electrophysiological studies, cells were plated on 8-mm glass coverslips (15,000 cells/well), and transfected using calcium phosphate with a mixture of pcDNA3-based expression plasmids harboring coding sequences for EGFP (10 ng/well), GABA_B_R1 (25 ng/well), GABA_B_R2 (25 ng/well), GIRK1 (25 ng/well), and either GIRK2a or GIRK2c (25 ng/well); experiments were conducted 16–24 h later. For immunoblotting experiments, cells were plated on 6-well plates (80,000 cells/well) and transfected using Lipofectamine 2000 reagent (Thermo Fisher Scientific; Waltham, MA); 2 μg of GIRK1, GIRK2a, GIRK2c or GIRK3 expression plasmids were used per well, and cells were harvested 48 h after transfection.

Primary cultures of hippocampal neurons were prepared as described^46^, with minor modifications. Briefly, hippocampi were extracted from neonatal (P1–4) pups and placed into an ice-cold modified Hank’s Balanced Salt Solution (HBSS, Sigma-Aldrich) containing 1 mM kynurenic acid, 12 mM MgCl_2_, 3 mM HEPES, and 5.5 mM D-glucose. The tissue was digested for 20 min with papain and DNAse I in the modified HBSS solution at 37 °C with occasional inversion. Tissue was then washed twice with modified HBSS, twice with modified HBSS plus 20% FBS, and twice with growth medium: Neural-basal A medium containing 2% B27 supplement, 0.5 mM Glutamax, and antibiotic/antimycotic (Thermo Fisher Scientific). Hippocampi were mechanically-dissociated in growth medium using trituration in 1 mL pipettes. Neurons were pelleted by centrifugation (150 × g, for 5 min at 4 °C), and plated onto 8-mm glass coverslips pre-coated with 0.0005% poly-L-lysine (Sigma-Aldrich) in 48-well plates. Neurons were maintained in culture at 37 °C/5%CO_2_, and half of the medium was replaced with fresh growth medium every 3–4 d. After 10 d in culture, neurons were infected with control or GIRK expression viruses; electrophysiological analysis or immunofluorescence microscopy was conducted 4–5 d after infection.

### Immunoblotting

Intact adult hippocampi and primary hippocampal cultures from wild-type and *Girk2* ^*−/−*^ mice were homogenized in RIPA lysis buffer containing Halt phosphatase and protease inhibitor cocktail (Thermo Fisher Scientific) and centrifuged at 4 °C for 20 min at 16,000 × g. The supernatant was taken and protein concentrations were determined by BCA assay. Equal amounts of protein from each sample were mixed with SDS sample buffer and heated at 85 °C for 10 min. Samples were then separated by 12% SDS-PAGE and transferred to nitrocellulose membranes. Membranes were blocked in 5% milk/PBS, incubated overnight at 4 °C with rabbit polyclonal antibodies directed against GIRK1 (1:200, Alomone Labs; Jerusalem, Israel), GIRK2 (1:200, Alomone Labs), GIRK3 (1 μg/mL, Frontier Institute Co.; Ishikari, Japan), or β-actin (0.27 μg/mL, Abcam; Cambridge, MA), and diluted in 5% milk/PBS/0.1% Tween 20. The custom GIRK2c antibody was generated and affinity-purified using a peptide corresponding to the unique 11-amino acid C-terminal domain (DVANLENESKV, Pacific Immunology Corp.; Ramona, CA). Membranes were washed with PBS/0.1% Tween 20 and incubated with donkey anti-rabbit (0.2 μg/mL, LI-COR Biotechnology; Lincoln, NE) or donkey anti-mouse (0.5 μg/mL; LI-COR) secondary antibodies. Blots were developed using the Odyssey infrared imaging system (LI-COR), and the integrated density of each band was measured using NIH ImageJ software (Bethesda, MD).

### Hippocampal organotypic culture and infection

Slice cultures were prepared as described^73^, with small modifications. Briefly, neonatal mice (7–9 d) were anesthetized with halothane in an induction chamber and decapitated. Brains were quickly removed and hippocampi dissected into ice-cold and oxygenated (95% O_2_/5% CO_2_) cutting medium: Minimal Essential Media (MEM) supplemented with 25 mM HEPES, 10 mM Tris-base, 10 mM glucose, and 3 mM MgCl_2_ (pH 7.2). Transverse hippocampal slices (400 μm) were cut using a tissue slicer (Stoelting Co.; Wood Dale, IL). Slices were placed onto transwell membrane inserts (Corning, Inc.; Corning, NY) in 6-well plates; each well was pre-filled with 1.6 mL of a 2:1 mixture of Basal Medium Eagle (Sigma) and Earle’s Balanced Salt Solution (Sigma) supplemented with (in mM): 20 NaCl, 5 NaHCO_3_, 0.2 CaCl_2_, 1.7 MgSO_4_ 48 glucose, 26.7 HEPES, penicillin (40,000 U/L) (Thermo Fisher Scientific), streptomycin (40 mg/L) (Thermo Fisher Scientific), insulin (1 mg/L) (Sigma-Aldrich), 0.5 ascorbic acid and 5% horse serum (Thermo Fisher Scientific) (pH 7.2). Slices were maintained in an incubator at 34 °C/5% CO_2_, and were fed 3 times per week by replacing half of the growth medium with fresh medium. Slices were infected with purified AAV viruses in a 1:50 dilution with 5% glycerol/10% sucrose PBS solution, using a Nanoject III injector (Drummond Scientific Company; Broomall, PA) one day after plating. Recordings were made 6–7 d post-infection.

### Electrophysiology in cultured cells

Coverslips containing transfected HEK cells or infected hippocampal neurons were transferred to a chamber containing a low-K^+^ bath solution (in mM): 130 NaCl, 5.4 KCl, 1 CaCl_2_, 1 MgCl_2_, 5.5 D-glucose, 5 HEPES/NaOH (pH 7.4). Neurons were visualized using an Olympus IX-70 microscope. Fire-polished borosilicate patch pipettes (3–5 MΩ) were filled with K-gluconate pipette solution (in mM): 140 K-gluconate, 2 MgCl_2_, 1.1 EGTA, 5 HEPES, 2 Na_2_-ATP, 0.3 Na-GTP, and 5 phosphocreatine (pH 7.2). Upon achieving whole-cell access, cells were held in voltage-clamp mode at −70 mV (with no correction for liquid junction potential). GIRK currents were measured in a high-K^+^ bath solution containing (in mM): 120 NaCl, 25 KCl, 1 CaCl_2_, 1 MgCl_2_, 5.5 D-glucose, 5 HEPES/NaOH (pH 7.4); GPCR agonists were dissolved in the high-K^+^ bath solution, which was then applied directly to the cell using a ValveLink 8.2 rapid perfusion system (AutoMate Scientific, Inc.; Berkeley, CA). Whole-cell currents were measured at room temperature with hardware (Axopatch-200B amplifier, Digidata 1322A) and software (pCLAMP v. 8.2) from Molecular Devices, LLC (Sunnyvale, CA). All currents were low-pass filtered at 2 kHz, digitized at 10 kHz, and stored on computer hard disk for subsequent analysis. Only those experiments in which the access resistances were stable and low (<18 MΩ) were included in the analysis. Peak and steady-state currents were measured for each experiment. Current activation and deactivation rates were extracted from 1-term Boltzmann/exponential fits of the recording segments corresponding to the onset and offset, respectively, of drug effect.

### Electrophysiology in organotypic slices

Organotypic hippocampal slices were cut out of the culture insert and positioned in a recording chamber superfused (2–2.5 mL/min) with an external solution containing (in mM): 125 NaCl, 2.5 KCl, 25 NaHCO_3_, 1.3 MgCl_2_, 2 CaCl_2_, 0.4 ascorbic acid and 10 glucose (pH 7.4) and bubbled with 95% O_2_/5% CO_2_. Kynurenic acid (2 mM) and picrotoxin (100 μM) were added to the bath solution to block ionotropic glutamate and GABA_A_ receptors, respectively. Bath and chamber temperatures were maintained at 29–30 °C using a TC344C Dual Automatic Temperature Controller (Warner Instruments; Hamden, CT). Whole-cell recordings (V_hold_ = −60 mV) were made from EGFP-positive CA1 pyramidal neurons using borosilicate pipettes (3–5 MΩ) filled with a K-gluconate pipette solution. All measured and command potentials factored in a junction potential (−15 mV) predicted using JPCalc software (Molecular Devices, LLC). Recordings were amplified with an EPC10 HEKA amplifier, filtered at 2 kHz, digitized at 5–10 kHz using Patchmaster 2x73.2 software (HEKA Elektronik; Bellmore, NY) and stored on hard disk. Stimulation was delivered with a cluster-type stimulating electrode (FHC, Bowdoin, ME) and a DS3 stimulator (Digitimer North America, LLC; Ft. Lauderdale, FL). Stimulating electrodes were placed in the *stratum radiatum* or *stratum lacunosum moleculare*, the dendritic fields that receive Schaffer Collateral (SC) and Perforant Path (PP) inputs, respectively. Stimuli of increasing intensity (0.2–25 mA) were applied at a 0.05 Hz frequency to establish maximal slow IPSC amplitude. Slow IPSCs evoked by SC and PP were made in each neuron, and the order of stimulation was counterbalanced across experiments. Only experiments with stable (<20% variation) and low series resistances (<30 MΩ) were analyzed.

### Immunofluorescence microscopy

Cultured hippocampal neurons were fixed with 4% paraformaldehyde and 0.4% sucrose in PBS for 10 min, permeabilized with 0.2% Triton X-100 in PBS for 10 min, washed with PBS, blocked with 10% normal goat serum in PBS for 1 h, and then incubated at 4 °C overnight with primary antibodies: pan-GIRK2 (1:500, Alomone Labs), PSD-95 (2 μg/mL, Thermo Fisher Scientific), MAP2 (1:1000, PhosphoSolutions; Aurora, CO) and CaMKIIα (1:1000, EMD Millipore; Billerica, MA). After washing 3–5 times in PBS, cells were incubated with secondary antibodies tagged with FITC (1:2000, Jackson ImmunoResearch Laboratories, Inc.; West Grove, PA) or Alexa-595/647 (2 μg/mL, Thermo Fisher Scientific) for 1 h at room temperature and protected from light. After 3 washes in PBS, coverslips were mounted onto glass slides for image acquisition. Fluorescent images were captured under a 40X or 60X (oil-immersion) objectives from a BX51W1 upright microscope with Disk Spinning Unit confocal system (Olympus; Center Valley, PA) and digital CCD camera (C10600-10B, Hamamatsu Photonic System Corp; Bridgewater, NJ). Images were processed and analyzed with Metamorph Advanced 7.7.7.0 (Molecular Devices) and NIH ImageJ software.

For quantification of GIRK2 fluorescence in dendritic processes, only infected neurons that were clearly separated from other infected cells were evaluated. MAP2 labeling was used to identify clearly the dendrites; 2–3 relatively straight segments (35–50 microns) of primary, secondary and tertiary dendrites exhibiting even MAP2 labeling from each neuron were selected for analysis. The primary dendrite fragments were located at least 20 microns from the soma. GIRK2 fluorescence intensity measurements within these processes were acquired following background subtraction, and data are presented as arbitrary units (AU). For GIRK2 isoform and PSD-95 co-localization analysis, the soma and surrounding area (20 microns from the edge of the soma) was removed from the image prior to analysis. Quantification was performed using the NIH ImageJ 1.29 plug-in, Puncta Analyzer. MAP2 labeling was used to delineate dendritic processes, and red (GIRK2) and green (PSD-95) channels were thresholded to highlight visible puncta without introduction of background noise. The percentage of co-localized GIRK puncta overlapping with total PSD-95 puncta was calculated for each analyzed neuron.

### Viral expression of GIRK2a and GIRK2c in dorsal CA1

CaMKIICre(+):*Girk2* ^*fl/fl*^ mice (7–8 wk) were placed in a stereotaxic device (David Kopf Instruments; Tujunga, CA) under isoflurane anesthesia. Microinjectors were created by affixing a 33-gauge stainless steel hypodermic tube within a shorter 26-gauge stainless steel hypodermic tube, and were attached to polyethylene-20 tubing affixed to 10 μL Hamilton syringes. Microinjectors were lowered bilaterally through burr holes in the skull to the dorsal CA1 (bregma: −2.20 mm AP, ±1.60 mm ML, −1.40 mm DV) and 400 nL of AAV8-hSyn-DIO-GIRK2a-IRES-EGFP, AAV8-hSyn-DIO-GIRK2c-IRES-EGFP, or AAV8-hSyn-DIO-mCherry control virus (~1.3 × 10^13^ viral particles/mL) was injected per side over 4 min using a Pump 11 Elite infusion pump (Harvard Apparatus; Holliston, MA). The syringe was left in place for 10 min following infusion to reduce solution backflow along the infusion track. Behavioral experiments were performed 2 wk after surgery to allow for full recovery and viral expression. The accuracy of viral targeting was assessed in all experiments using fluorescence confocal microscopy. Images were acquired with MetaMorph Advanced Acquisition v. 7.7.7.0 software. GIRK-dependent signaling in CA1 pyramidal neurons expressing GIRK2a or GIRK2c, indicated by EGFP expression, was assessed by measuring baclofen-induced somato-dendritic currents in acutely isolated slices, as described^6^.

### Fear conditioning

To most effectively isolate the contribution of the hippocampus to context fear learning, we utilized a trace fear conditioning protocol^45^. In brief, mice were acclimated to and handled within the testing room for 2 d prior to testing. For the training session (Day 1), mice were placed in fear conditioning chambers (Med Associates, Inc.; St Albans, VT) with metal bar floors and white wall insert that were cleaned with 75% ethanol. A 30-s white noise (65 dB/3 kHz) was used as the conditioned stimulus (CS, cue), and a 2-s footshock (0.50 mA) was used as the unconditioned stimulus (US). Training consisted of 2 CS-US pairings in which the US was delivered 30 s after the end of the CS; CS presentations were separated by 90 s (7.5 min total conditioning time). On Day 2, the level of contextual fear learning was assessed by returning mice to the training chambers and measuring freezing behavior for 5 min. On Day 3, the chambers were reconfigured (*i.e*., floor bars were covered with a plastic insert and white wall inserts were replaced with black tent inserts to alter both the color and shape of the chamber) and cleaned with 0.1% acetic acid. Mice were then assessed for freezing behavior in response to 2 × 3-min CS presentations. Freezing was assessed by Video Freeze software (Med Associates, Inc.) and data are presented as percent time spent freezing.

### Statistical analysis

Data are presented throughout as the mean ± SEM. Statistical analyses were performed using Prism 6 or 7 (GraphPad Software, Inc.; La Jolla, CA). Data were analyzed with Student’s two-tailed *t*-test, 1-way ANOVA, 2-way ANOVA, or 2-way ANOVA with repeated measures, as appropriate. Tukey, Holm-Sidak, Dunnett’s, and Bonferroni multiple comparison *post hoc* tests were used, if justified. Differences were considered significant if *P* < 0.05.

### Data availability

The datasets generated during and/or analyzed during the current study are available from the corresponding author on reasonable request.

## Electronic supplementary material

Supplementary Information
