# Supplementary material for: GIRK2 splice variants and neuronal G protein-gated K+ channels: implications for channel function and behavior
Source: Sci Rep. 2017 May 9;7:1639. doi: 10.1038/s41598-017-01820-2 (PMC5431628; doi:10.1038/s41598-017-01820-2)
Supplement: Supplementary file 1 — Supplementary Information [file 41598_2017_1820_MOESM1_ESM.pdf]

SUPPLEMENTARY INFORMATION

GIRK2 splice variants and neuronal G protein-gated K^+ channels: implications for channel function and behavior

Ezequiel Marron Fernandez de Velasco[1+], Lei Zhang[1+], Baovi Vo[1+], Megan Tipps[1], Shannon Farris[2], Zhilian Xia[1], Allison Anderson[1], Nicholas Carlblom[1], C. David Weaver[3], Serena M. Dudek[2], Kevin Wickman[1*]

[1] University of Minnesota, Department of Pharmacology, Minneapolis, MN 55455 USA
[2] National Institute of Environmental Health Sciences, Research Triangle Park, NC 27709 USA
[3] Vanderbilt University, Department of Pharmacology, Nashville, TN 37235 USA
* wickm002@umn.edu
[+] these authors contributed equally to this work

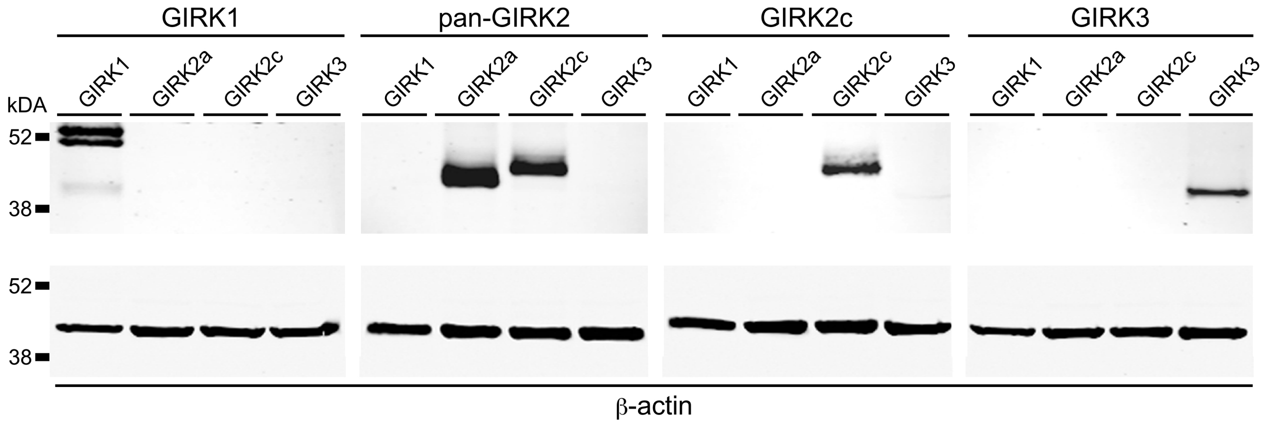

Supplementary Figure S1. Validation of GIRK2c-specific antibody specificity. Representative immunoblots (from 3 independent experiments) demonstrating GIRK2c-specific antibody specificity in HEK cells expressing GIRK1, GIRK2a, GIRK2c, or GIRK3. Blots were probed with GIRK1, pan-GIRK2, GIRK2c, or GIRK3 antibodies, as well as a β-actin antibody. Note that the GIRK1 antibody recognizes both core and glycosylated versions of the GIRK1 protein[19], resulting in a doublet in immunoblotting experiments.

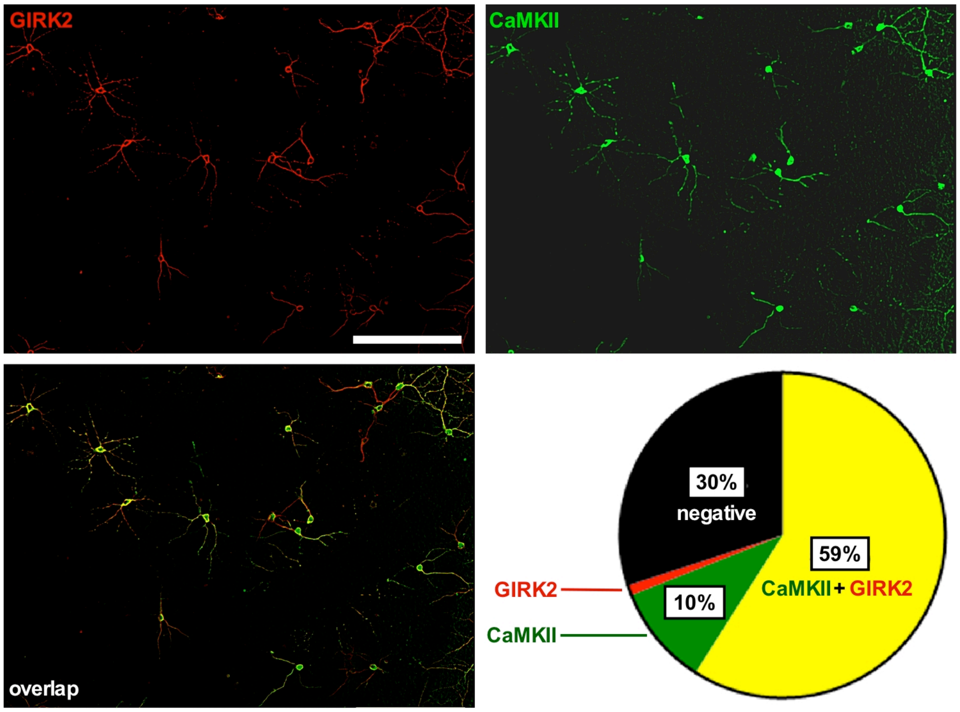

Supplementary Figure S2. Viral expression of GIRK2 cultured hippocampal pyramidal neurons. Representative images showing recombinant GIRK2 (red) and endogenous CaMKII (green) immunolabeling, and their overlap, in *Girk2*$^{-/-}$ hippocampal neurons infected with the AAV8-CaMKII-GIRK2a-IRES-EGFP virus; scale bar – 200 microns. The pie chart shows that 59% of neurons were labeled with both GIRK2 and CaMKII antibodies. A significant fraction of neurons did not show detectable labeling for either GIRK2 or CaMKII (negative, 30%), and the remainder exhibited detectable labeling for only CaMKII (10%) or GIRK2 (1%).

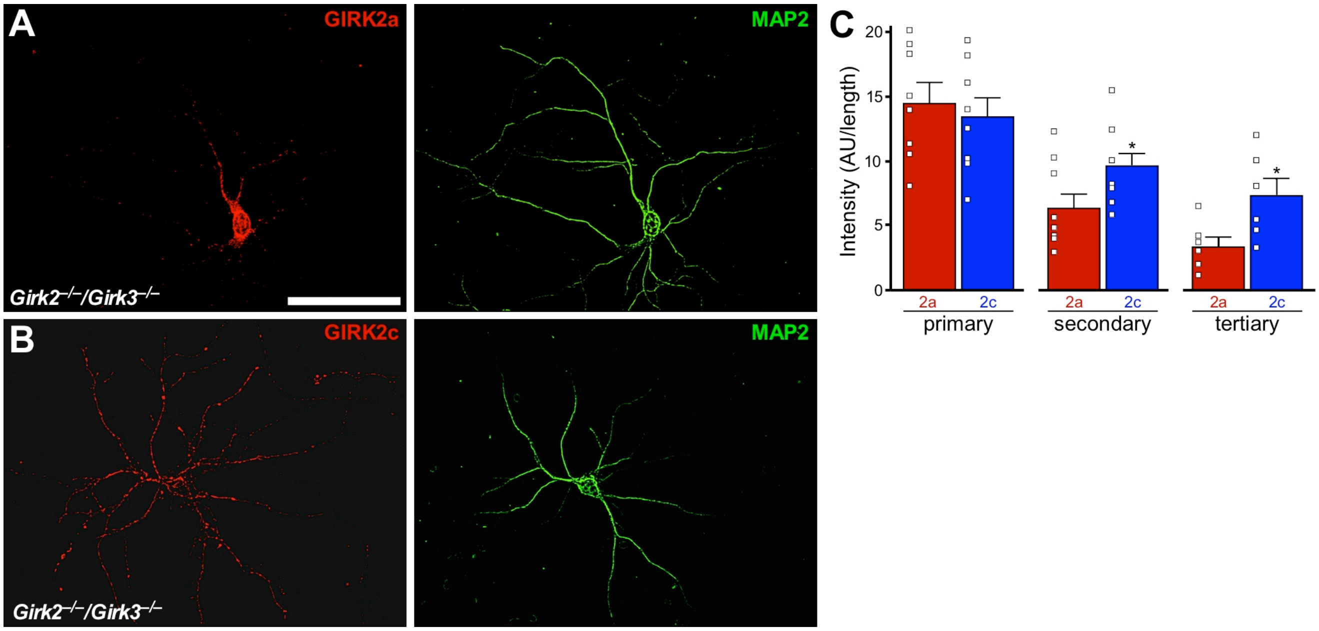

Supplementary Figure S3. GIRK2a and GIRK2c distribution in *Girk2*$^{-/-}$/*Girk3*$^{-/-}$ pyramidal neurons.

A,B. Representative images of recombinant GIRK2a (A) and GIRK2c (B) labeling in hippocampal pyramidal neurons from *Girk2*$^{-/-}$/*Girk3*$^{-/-}$ mice; scale bar – 50 microns.

C. Quantification of GIRK2a and GIRK2c labeling in dendrites from infected *Girk2*$^{-/-}$/*Girk3*$^{-/-}$ pyramidal neurons. GIRK2 fluorescence intensity was measured in 2-3 primary (t_{14}=1.0, P=0.60; n=8 segments/isoform), secondary (t_{16}=2.2, *P<0.05; n=7-8 segments/isoform), and tertiary (t_{10}=2.5, *P<0.05; n=6 segments/isoform) dendritic segments from 3 different neurons expressing each subunit. Fluorescence intensity is expressed as arbitrary units (AU) normalized to segment length. Only GIRK2 labeling that overlapped directly with the dendritic segment (identified by MAP2 labeling) was quantified in this analysis.

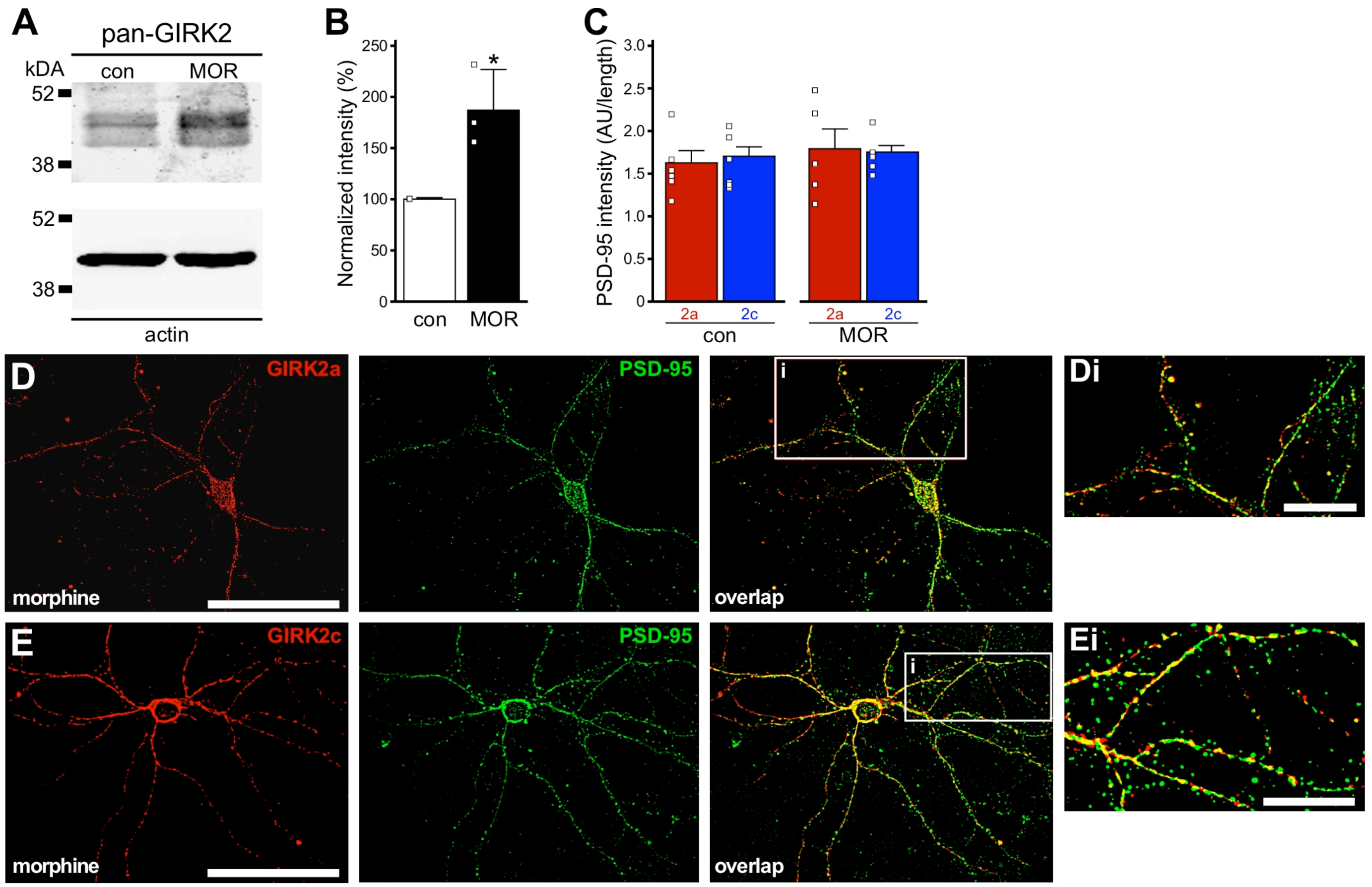

Supplementary Figure S4. Effect of morphine on GIRK2 and PSD-95 in hippocampal cultures

A. Representative immunoblots (from 3 independent experiments) showing total GIRK2 and β-actin protein levels in wild-type hippocampal cultures under control (con) conditions, and following a 20-h morphine treatment (MOR, 100 μM).

B. Summary of immunoblotting data showing the impact of morphine treatment on total GIRK2 protein level in wild-type hippocampal cultures (t_4=3.8, *P<0.05; n=3 independent experiments). GIRK2 protein level was normalized to the level of β-actin in each sample.

C. Quantification of PSD-95 immunolabeling intensity at baseline (con) and following morphine treatment, in dendrites from *Girk2*$^{-/-}$ pyramidal neurons expressing GIRK2a or GIRK2c (n=5-6 per condition and isoform). For this analysis, dendrites were identified by MAP2 labeling (not shown) and the soma and surrounding area (20 microns from the edge of the soma) were removed from the image prior to analysis. PSD-95 labeling intensity is expressed as arbitrary units (AU) normalized to dendritic segment length. No significant effect of isoform ($F_{1,16}$=0.004, P=0.95) or treatment ($F_{1,16}$=0.40, P=0.54) was detected, nor was there an interaction between isoform and treatment ($F_{1,16}$=0.15, P=0.70).

D,E. Representative images showing GIRK2 and PSD-95 immunolabeling, and their overlap, in a *Girk2*$^{-/-}$ hippocampal pyramidal neurons expressing GIRK2a (D) or GIRK2c (E), after morphine treatment; scale bar: 50 microns. Insets (Di and Ei) highlight the overlap between PSD-95 and GIRK2 in dendrites; scale bar – 5 microns.

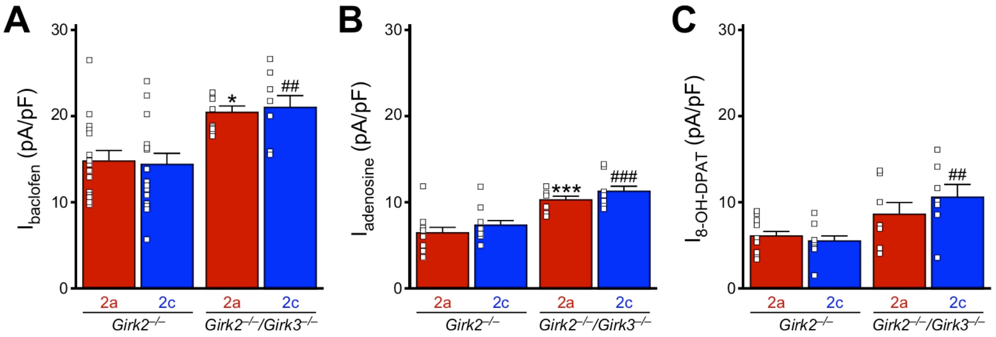

Supplementary Figure S5. GPCR-GIRK currents in *Girk2*$^{-/-}$/*Girk3*$^{-/-}$ neurons expressing GIRK2a or GIRK2c

A. Summary of baclofen-induced, steady-state current densities (pA/pF) in *Girk2*$^{-/-}$/*Girk3*$^{-/-}$ pyramidal neurons expressing GIRK2a or GIRK2c (n=7-8/isoform). Data from *Girk2*$^{-/-}$/*Girk3*$^{-/-}$ neurons was compared with respective data from *Girk2*$^{-/-}$ neurons, taken from Figure 5. A main effect of genotype was observed ($F_{1,42}$=20.0, P<0.001), but there was no main effect of isoform ($F_{1,42}$=0.004, P=0.95) or interaction between genotype and isoform ($F_{1,42}$=0.1, P=0.72). Symbols: *P<0.05 vs. GIRK2a (*Girk2*$^{-/-}$); ##P<0.01 vs. GIRK2c (*Girk2*$^{-/-}$).

B. Summary of adenosine-induced, steady-state current densities (pA/pF) in *Girk2*$^{-/-}$/*Girk3*$^{-/-}$ pyramidal neurons expressing GIRK2a or GIRK2c (n=7-8/isoform). Data from *Girk2*$^{-/-}$/*Girk3*$^{-/-}$ neurons was compared with respective data from *Girk2*$^{-/-}$ neurons, taken from Figure 5. A main effect of genotype was observed ($F_{1,30}$=34.0, P<0.001), but there was no main effect of isoform ($F_{1,30}$=2.0, P=0.17) or interaction between genotype and isoform ($F_{1,30}$=0.01, P=0.92). Symbols: ***P<0.001 vs. GIRK2a (*Girk2*$^{-/-}$); ###P<0.001 vs. GIRK2c (*Girk2*$^{-/-}$).

C. Summary of 8-OH-DPAT-induced, steady-state current densities (pA/pF) in *Girk2*$^{-/-}$/*Girk3*$^{-/-}$ pyramidal neurons expressing GIRK2a or GIRK2c (n=7/isoform). Data from *Girk2*$^{-/-}$/*Girk3*$^{-/-}$ neurons was compared with respective data from *Girk2*$^{-/-}$ neurons, taken from Figure 5. A main effect of genotype was observed ($F_{1,31}$=14.3, P<0.001), but there was no main effect of isoform ($F_{1,31}$=0.6, P=0.45) or interaction between genotype and isoform ($F_{1,31}$=1.5, P=0.23). Symbols: ##P<0.01 vs. GIRK2c (*Girk2*$^{-/-}$).
